# Employing Macrophage-Derived Microvesicle for Kidney-Targeted Delivery of Dexamethasone: An Efficient Therapeutic Strategy against Renal Inflammation and Fibrosis

**DOI:** 10.7150/thno.33520

**Published:** 2019-07-09

**Authors:** Tao-Tao Tang, Lin-Li Lv, Bin Wang, Jing-Yuan Cao, Ye Feng, Zuo-Lin Li, Min Wu, Feng-Mei Wang, Yi Wen, Le-Ting Zhou, Hai-Feng Ni, Ping-Sheng Chen, Ning Gu, Steven D. Crowley, Bi-Cheng Liu

**Affiliations:** 1 Institute of Nephrology, Zhong Da Hospital, School of Medicine, Southeast University, Nanjing, China; 2 State Key Laboratory of Bioelectronics, Jiangsu Key Laboratory for Biomaterials and Devices, School of Biological Sciences and Medical Engineering, Southeast University, Nanjing, China; 3 Division of Nephrology, Department of Medicine, Duke University and Durham VA Medical Centers, Durham, North Carolina, United States

**Keywords:** renal inflammation, renal fibrosis, macrophage-derived microvesicles, dexamethasone, drug delivery

## Abstract

Although glucocorticoids are the mainstays in the treatment of renal diseases for decades, the dose dependent side effects have largely restricted their clinical use. Microvesicles (MVs) are small lipid-based membrane-bound particles generated by virtually all cells. Here we show that RAW 264.7 macrophage cell-derived MVs can be used as vectors to deliver dexamethasone (named as MV-DEX) targeting the inflamed kidney efficiently.

**Methods**: RAW macrophages were incubated with dexamethasone and then MV-DEX was isolated from the supernatants by centrifugation method. Nanoparticle tracking analysis, transmission electron microscopy, western blot and high-performance liquid chromatography were used to analyze the properties of MV-DEX. The LC-MS/MS was applied to investigate the protein compositions of MV-DEX. Based on the murine models of LPS- or Adriamycin (ADR)-induced nephropathy or in-vitro culture of glomerular endothelial cells, the inflammation-targeting characteristics and the therapeutic efficacy of MV-DEX was examined. Finally, we assessed the side effects of chronic glucocorticoid therapy in MV-DEX-treated mice.

**Results**: Proteomic analysis revealed distinct integrin expression patterns on the MV-DEX surface, in which the integrin α_L_β_2_ (LFA-1) and α_4_β_1_ (VAL-4) enabled them to adhere to the inflamed kidney. Compared to free DEX treatment, equimolar doses of MV-DEX significantly attenuated renal injury with an enhanced therapeutic efficacy against renal inflammation and fibrosis in murine models of LPS- or ADR-induced nephropathy. *In vitro*, MV-DEX with about one-fifth of the doses of free DEX achieved significant anti-inflammatory efficacy by inhibiting NF-κB activity. Mechanistically, MV-DEX could package and deliver glucocorticoid receptors to renal cells, thereby, increasing cellular levels of the receptor and improving cell sensitivity to glucocorticoids. Notably, delivering DEX in MVs significantly reduced the side effects of chronic glucocorticoid therapy (e.g., hyperglycemia, suppression of HPA axis).

**Conclusion**: In summary, macrophage-derived MVs efficiently deliver DEX into the inflamed kidney and exhibit a superior capacity to suppress renal inflammation and fibrosis without apparent glucocorticoid adverse effects. Our findings demonstrate the effectiveness and security of a novel drug delivery strategy with promising clinical applications.

## Introduction

Glucocorticoids are widely prescribed medications used to treat a variety of renal diseases with an inflammatory component 1. However, systemic glucocorticoid therapy is associated with multiple serious adverse effects in a dose-dependent manner (for example, infection, hypertension, hyperglycemia, concentric obesity, osteoporosis, etc.) 1,2. Such undesirable effects greatly limit their clinical applications, thus developing a targeted approach that can improve tissue-specific delivery and drug bioavailability is urgently needed.

Extracellular vesicles (EVs), such as exosomes and microvesicles, are host-cell derived membrane particles with a natural mode of intercellular communication 3-6. The capacity of EVs has spurred a renewed interest in their utility as delivery vehicles for various therapeutic agents 6-8. Recent studies show that EVs can function as efficient carriers of chemotherapeutic drugs 9-11, RNA drugs 12-14 and anti-inflammatory drugs 15,16. Whether EVs are suitable for glucocorticoid delivery remains unknown.

Delivery and retention of drugs in the kidney is challenging because of difficulty in targeting specific cell populations and passing glomerular filtration barriers 17,18. Although decoration of EVs with targeting peptide is likely to provide novel solutions 13,19, such modification can provoke unintended immune responses, especially during chronic treatments. Given that there is an abundance of membrane proteins on the surface of EVs, we can take advantage of that to enhance their specific cell interactions 20. Recently, Yuan et al. demonstrated that naïve exosomes derived from macrophages can interact with inflamed endothelium through exosomal adhesion molecules 21. Considering the presence of inflammation in most renal diseases 22,23, we investigated whether macrophage-derived EVs can utilize their surface proteins and target a therapeutic cargo into the kidney parenchyma following an inflammatory insult.

To evaluate the therapeutic efficacy of dexamethasone-loaded macrophage-derived microvesicles (MV-DEX) we generated, murine models of LPS- or Adriamycin (ADR)-induced nephropathy were applied, in which routine systemic DEX therapy has proven to be effective 24,25. In the present study, we found that macrophage-derived MVs efficiently deliver DEX into the inflamed kidney and exhibit a superior capacity to suppress renal inflammation without apparent glucocorticoid adverse effects when compared to free DEX treatment. Mechanistically, the glucocorticoid receptor in the MVs can be transferred to recipient cells and facilitate the anti-inflammatory efficacy. To our knowledge, this is the first report of EVs utilized as nanocarriers for drug delivery in the treatment of renal diseases.

## Methods

### Cell culture

RAW 264.7 cells were from American Type Culture Collection (ATCC) and cultured in RPMI (Gibco) with 10% FBS (Gibco) and 1% penicillin- streptomycin (Invitrogen). For downregulation of glucocorticoid receptor (GR), transfections were performed using Lipofectamine RNAimax (Invitrogen) with GR-targeted siRNA or control siRNA (GenePharma). For tracing the GR, transfections were performed with recombinant lentivirus for Nr3c1-RFP (Genechem) following the manufacturer's instructions. Primary cultures of mouse glomerular endothelial cells (GECs) were prepared as previously described 26. In brief, kidneys were removed, decapsulated, minced into 1 mm^3^ pieces, and were gradually sieved with stainless steel sieves to get the glomeruli. Separated glomeruli were washed with DMEM and subsequently treated with 0.1% collagenase type Ⅱ (Sigma-Aldrich) in DMEM at 37℃ for 40 minutes. During the digestion period, the solution was vortexed every 10 minutes. Afterwards, the cell suspension was washed in Hanks buffered salt solution containing 10% fetal calf serum (Invitrogen), and subsequently resuspended in endothelial cell culture medium (ScienCell) supplemented with 60 mg/ml endothelial cell growth supplement (ScienCell), 5 U/ml heparin, 10% FBS (Gibco), and 1% penicillin- streptomycin (Invitrogen). The cells were cultured on fibronectin (ScienCell)-coated plates. On days 5 to 7 after the initial seeding, outgrowths of GECs were visible and were verified by immunostaining against CD31 (endothelial cell), nephrin (podocyte) and α-SMA (mesangial cell) (Figure S12).

### Preparation and characterization of durg-packaging MVs

RAW 264.7 cells were treated with DEX (30 μmol/L, Sigma-Aldrich) for 16 h without FBS, and then supernatants were collected for MVs isolation as described before 9. Briefly, supernatants were first centrifuged at 2000×g for 20 min to get rid of cells and debris. Subsequently, supernatants were further centrifuged at 16,500×g for 30 min to get MVs. The pellet was washed one time and resuspended in sterile PBS or culture medium for the following experiments. The size distribution and quantity were detected by nanoparticle tracking analysis (ZetaView PMX 110, Particle Metrix). The morphology was examined by transmission electron microscopy (TEM) (H-7650, HITACHI). Surface markers of isolated MVs were detected by Western blot. For treatment of MV-DEX with proteinase K 12, purified MV-DEX was incubated (37 °C, 30 min) with 5 mg/ml of proteinase K (Sigma-Aldrich) followed by heat inactivation (60 °C, 20 min). For blockade of integrins, purified MV-DEX was incubated with 20 μg/ml of anti-integrin β2 antibodies (555280, BD Biosciences) or anti-VLA-4 antibodies (BE0071, Hölzel Diagnostics) or rat IgG overnight at 4 °C.

### Animal models and therapeutic experiments

For LPS-induced nephropathy, all mice (8 weeks of age; C57BL/6) were injected intraperitoneally with either LPS (10 μg/g, Sigma-Aldrich) or sterile PBS at 0- and 24-hour time points as previously described 27,28. At the 12-hour time point after the initial LPS injection, mice were injected intravenously with vehicle, free DEX (0.5 mg/kg, Sigma-Aldrich), or MV-DEX (with 0.5 mg/kg DEX), respectively. An additional treatment was administered at the 36-hour time point. Mice were euthanized at 48 hours post- LPS treatment. For ADR-induced nephropathy, all mice (8 weeks of age; C57BL/6) were injected intravenously with either ADR (18 mg/kg, Sigma-Aldrich) or sterile PBS as previously described 25. Free DEX (0.5 mg/kg), MV-DEX (with 0.5 mg/kg DEX), or vehicle was administered by tail vein starting at day 7 post-ADR treatment, respectively, and continued every 2 days. Mice were euthanized at 3 weeks post-ADR treatment. For downregulation of GR in the kidney, mice were treated daily by intraperitoneal injections of RU486 (20 mg/kg, Sigma-Aldrich) or vehicle. After 7 days, LPS-treated mice were established as described above. Urine albumin and creatinine in the same samples, and serum creatinine levels were measured with commercial kits according to manufacturer's instructions (Jiancheng). The urine protein excretion rate was expressed as the ratio of albumin to creatinine.

### Labelling of MV-DEX

RAW 264.7 cells were labeled with lipophilic dyes DID (Invitrogen), PKH 26 (Sigma-Aldrich), or PKH 67 (Sigma-Aldrich) according to the manufacturer's protocol, respectively, and the free dye was washed away with PBS three times. RAW 264.7 cells were then stimulated with DEX and MVs released from DID-, PKH26-, or PKH67-stained cells were isolated as described above. This method can remove the free dye in pellet to the greatest extent.

### *In vivo* biodistribution of MV-DEX

For LPS-treated mice, DID-labeled MV-DEX (1×10^10^) was injected intravenously at 24 hours post- LPS treatment. For ADR-treated mice, DID-labeled MV-DEX (1×10^10^) was injected intravenously at 7 days post-ADR treatment. Sham mice were injected with sterile PBS. All animals were sacrificed at 24 hours after administration, and organs (heart, lung, liver, spleen and kidney) were excised and imaged using the IVIS Spectrum imaging system (PerkinElmer) 29. The average radiance (photons·s^-1^·cm^-2^·sr^-1^) of each kidney was obtained under the same conditions for all experimental groups. In addition, the specific organs were also obtained and mounted in OCT compound and frozen. Sectioned tissue was stained with DAPI nuclear stain, and images were then captured using confocal microscope (FV1000, Olympus). Images were quantified by counting the number of nuclei within DID-labeled MV-DEX surrounding it and dividing by the total number of nuclei, in six random tissue sections per organ. In the kidney, accumulation of DID-labeled MV-DEX was quantified in both glomeruli and tubulointerstitium.

### Cellular uptake of MV-DEX *in vitro*

PKH 26 or PKH 67-labeled MVs (5×10^7^) were incubated with GECs that administered with or without LPS for 3 or 6 h, respectively, and then PKH 26-positive cells were observed under confocal microscope, while PKH 67-positive cells were analyzed by flow cytometry. The number of PKH 26-labeled MVs (red dots) in each cell were counted in at least 10 randomly selected fields from each well and results were averaged for each cell.

### Proteomic analysis

Mass spectrometry analyses of MV-DEX were performed at GeneChem using 200 μg of MV protein. Samples extracted with SDS- and DTT- based buffer were precipitated using acetone. After repeated rinsing with acetone, the protein pellets were freeze- dried and denatured using 8 M urea, reduced using 10 mM DTT, and alkylated using 100 mM iodoacetamide. This was followed by digestion with trypsin for 16 h at 37 °C. For the analysis, approximately 10 μg of each sample was analysed by reversed phase nano- liquid chromatography-tandem mass spectrometry (LC-MS/MS) (EASY-nLC coupled to Q Exactive Plus, Thermo Scientific). In brief, peptides were separated using a 50-μm inner diameter C18 column (Thermo Scientific) at a flow rate of 300 nL/min. Precursor mass spectra were recorded in a 350-1,800 *m/z* mass range at 70,000 resolution, and 1,7500 resolution for fragment ions. Data obtained were analysed using Proteome Discoverer 2.1 (Thermo Scientific) against the UniProt database using the following parameters: maximum number of missed cleavages, 2; precursor tolerance, 20 ppm; dynamic modification: oxidation (M) and N-terminal protein acetylation, deamidated (NQ); static modification: carbamidomethyl (C); and false discovery rate, <0.01.

### Kidney histology and quantification

For histology analysis, kidneys were fixed with 4% formaldehyde, embedded in paraffin, and sectioned to 4 μm thickness. Renal sections were used for PAS or Masson's trichrome staining. To blindly evaluate glomerulosclerosis index, 50 sequential glomeruli per animal were assessed on Masson's trichrome-stained sections, and semiquantitatively scored as follows: 0=none, 1=0%-25%, 2=25%-50%, 3=50%-75%, and 4=>75% 30. Sclerosis index is the average. To evaluate interstitial fibrosis, at least 10 randomly selected fields for each kidney on Masson's trichrome-stained sections were assessed. Tissue fibrosis as defined by blue staining was scored by three experienced observers masked to experiment conditions. The percentage of blue staining in each image was calculated, and the mean values of the fibrosis scores were reported. For TEM, electron microscopic sample handling and detection were performed by electron microscopic core lab at Southeast University. The foot process width was assessed using Image J. Three glomeruli were randomly selected from each animal and five electron micrographs were taken in each glomerulus. Histology of kidney was scored by an observer masked to the treatment groups.

### Immunohistochemistry and immunofluorescence staining

For IHC, formalin-fixed and paraffin-embedded kidney sections were incubated with primary antibodies against CD68 (ab955, Abcam), neutrophil (ab2557, Abcam), or CD3 (ab16669, Abcam), and then analyzed using streptavidin peroxidase detection system (Maixin) according to the manufacturer's protocol. DAB (Maixin) was used as an HRP-specific substrate. Quantitative analysis of the number of positive cells was performed under original magnification (×400) in 40 randomly selected fields per mice in a blinded fashion. Immunofluorescence staining of formaldehyde-fixed cells was performed with primary antibody against NF-κB p-p65 (3033, CST), followed by incubation with a secondary antibody (Invitrogen, USA). Cell nuclei were stained with DAPI. Immunostained samples were visualized under a confocal microscope. Images were quantified by counting the number of positive nuclei and dividing by the total number of nuclei.

### High-performance liquid chromatography

MV-DEX was firstly processed by lysis buffer, proteinase K, phenylmethylsulphonyl fluoride and DNase I according to previous description 9. The concentration of DEX was measured by HPLC (Aglient 1200) with C18 column (250 mm × 4.6 mm). Acetonitrile and water (v/v=4:6) were used as the mobile phase. The column temperature and the detection wavelength were set as 25 °C and 240 nm with flow rate at 1.0 mL·min^-1^.

### ELISA detection

The TNF and IL-6 levels in the mice serum, cell-free supernatants, and renal tissue lysates were measured using ELISA kits (TNF, R&D Systems; IL-6, BD Systems; CTX-I, Immuno Diagnostic Systems; PⅠNP, Immuno Diagnostic Systems), according to the manufacturer's instructions.

### Luciferase Reporter Gene Assay

GECs were transfected with NF-κB luciferase reporter using Lipofectamine 2000 reagent (Invitrogen) and NF-κB luciferase reporter kit (BPS Bioscience). The luciferase activity was measured by BPS Dual Luciferase Assay System, according to the manufacturer's instructions. The results were normalized by the transfection efficiency calculated by the Renilla luminescence from the negative reporter plasmid.

### Quantitative real-time PCR assay

The total RNA from cells or renal cortex tissues was extracted using the TRIzol (Takara), and cDNA was then synthesized using PrimeScript RT reagent kit (Takara). Real-time RT-PCR was performed using a 7300 real-time PCR System (Applied Biosystems). The results were analyzed using the comparative cycle threshold (ΔΔCt) method. All the primer sequences were listed in the Supplementary Table 2.

### Western blot

The protein lysates from the cells and kidney tissues were prepared following standard protocols, and the protein content was determined using the BCA protein assay kit (ThermoFisher). And then the proteins samples were separated by Bis-Tris Gel (Invitrogen) and transferred onto PVDF membranes (Millipore) using a wet- transfer system. Membranes were blocked in 5% BSA in TBS-T for 1 h at room temperature and were incubated with primary antibodies overnight at 4 °C. Then membranes were washed and incubated with secondary horseradish peroxidase-conjugated antibodies for 2 h at room temperature, and the signals were detected using an ECL advanced system (GE Healthcare). Intensity values expressed as the relative protein expression were normalized to β-actin. Primary antibodies used were anti-CD68 (ab955, Abcam); anti-F4/80 (ab6640, Abcam); anti-CD206 (ab64693, Abcam); anti-actinin 4 (15145, Cell Signaling Technology); anti-integrin α4 (8440, Cell Signaling Technology); anti-integrin β1 (4706, Cell Signaling Technology); anti-integrin αL (ab186873, Abcam); anti-integrin β2 (ab185723, Abcam); anti-NF-κB p65 (8242, Cell Signaling Technology); anti-NF-κB p-p65 (3033, Cell Signaling Technology); anti-GR (12041, Cell Signaling Technology); anti-β-actin (ab8226, Abcam). Secondary HRP-conjugated antibodies used were anti-mouse IgG, anti-rabbit IgG, and anti-rat IgG (Abcam).

### Statistical analysis

Data were expressed as the mean ± SD. Statistical analysis were performed using t test or one-way ANOVA. P < 0.05 was considered statistically significant.

## Results and Discussion

### Preparation and characterization of RAW 264.7 macrophages-derived DEX-packaging MVs

**Figure 1:**
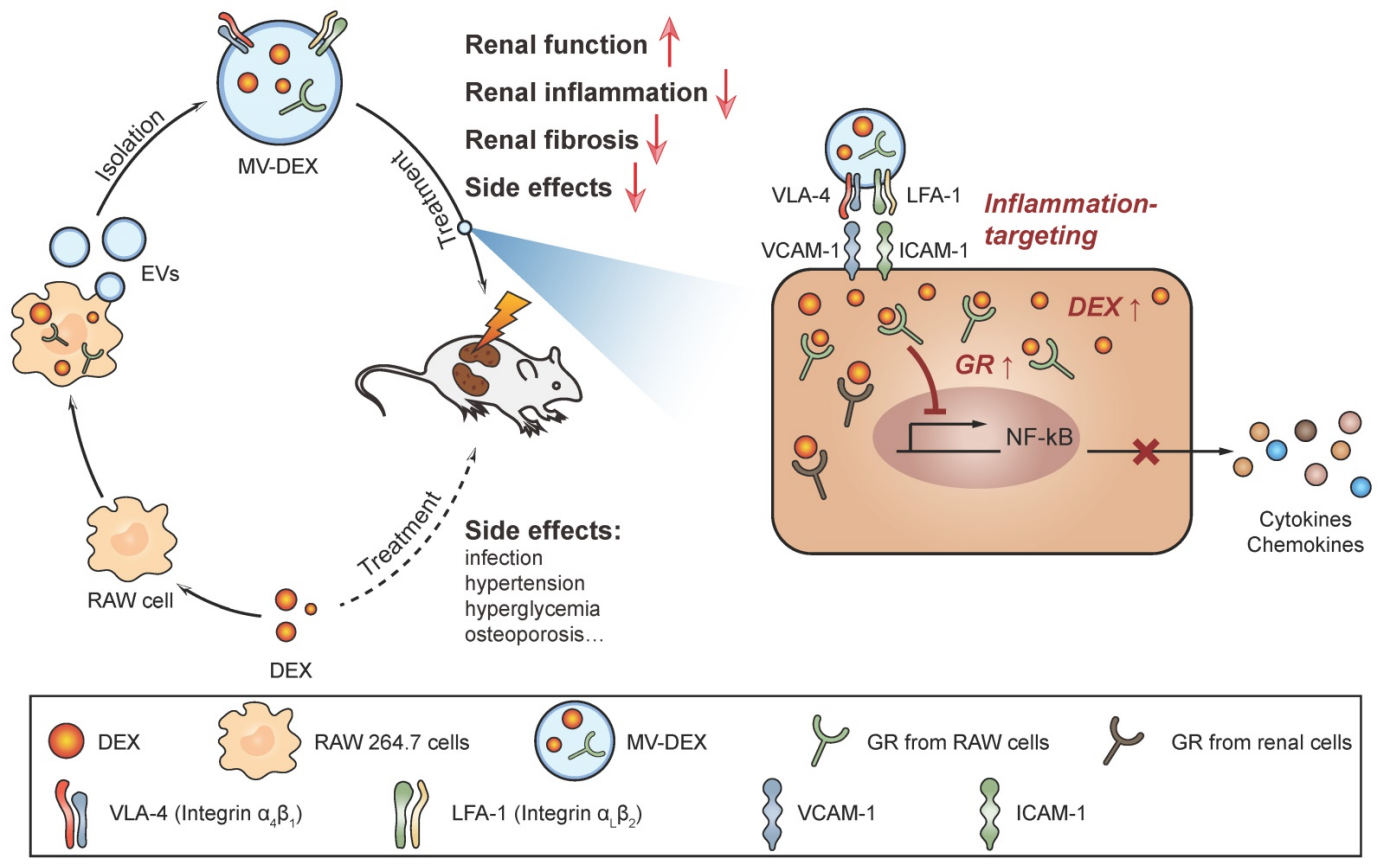
** Schematic illustration of the preparation of MV-DEX and the therapeutic effects of MV-DEX against renal diseases.** RAW macrophages were treated with DEX and then supernatants were collected for MV isolation by centrifugation. MV-DEX treatment improved renal function, alleviated renal inflammation and fibrosis, and reduced side effects to a greater magnitude than free DEX. Mechanistically, MV-DEX efficiently targeted inflamed kidney through LFA-1 and VLA-4 on the surface. Further, MV-DEX increased the cellular levels of the GR and facilitated the entry of DEX into the cells.

Cells release heterogeneous vesicles of different sizes and intracellular origins, including exosomes and microvesicles. Recent study finds that the epidermal growth factor receptors in microvesicles exhibit a higher level of functional similarity with the originating cells than those in exosomes 31. This indicates that the function of membrane proteins on microvesicles is not affected when budding from the plasma membrane. Meanwhile, the collection and purification of microvesicles are more feasible relative to exosomes. Thus, we employed microvesicles as the carrier for the delivery of DEX. To develop DEX-packaging MVs, RAW macrophages were incubated with DEX and then MVs were isolated from the supernatants by centrifugation method, as shown in Figure 1.

**Figure 2:**
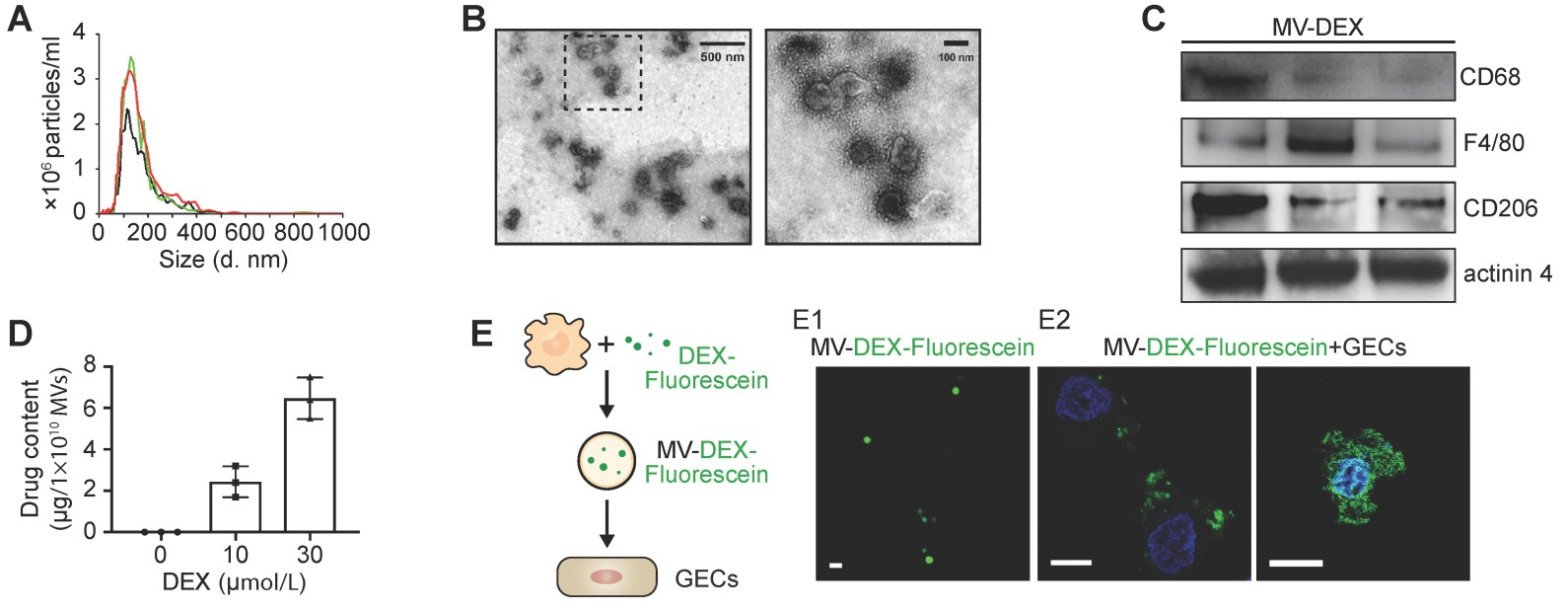
** Characterization of RAW 264.7 macrophages-derived DEX-packaging MVs.** (**A**) Size distribution analysis of MV-DEX by nanoparticle tracking analysis. (**B**) Transmission electron microscopy images of MV-DEX showed double-membrane vesicles. Boxed area is enlarged in the right. (**C**) Markers of macrophage (CD68, F4/80 and CD206) and microvesicles (actinin 4) were confirmed in MV-DEX by Western blot. (**D**) HPLC analysis revealed the concentrations of DEX in MVs were dependent on the applied drug doses. (**E**) RAW cells were incubated with 30 μmol/L fluorescein-DEX. MVs were isolated and observed under confocal microscopy. DEX was shown as the green fluorescent (E1) (scale bar, 1 μm). GECs were incubated with fluorescein-DEX packaging MVs for 12 h and then observed under confocal microscopy (E2) (scale bar, 10 μm). n=3. Data are presented as mean ± SD.

The collected EVs were characterized by several parameters, including size (Figure 2A), morphology (Figure 2B), plasma membrane origin (Figure 2C, Figure S1A), drug concentration (Figure 2D, Figure S1B) and stability (Figure S1C). Nanoparticle tracking analysis revealed that the size distribution of the EVs ranged from 15 to 500 nm with a mean diameter of 140.7±4.8 nm (Figure 2A). Transmission electron microscopy showed typical morphology as published 8-10 (Figure 2B). Macrophage markers including CD68, F4/80 and CD206, and microvesicle marker actinin-4 32 were identified by Western blot (Figure 2C), while the expression of exosomal markers (Alix and TSG101) was very low in the pellets (Figure S1A), indicating that the vesicles we collected were mainly microvesicles. HPLC analysis showed that the different concentrations of DEX in MVs were dependent on the applied drug doses (Figure 2D, Figure S1B). We employed fluorescein-DEX to further determine whether the drug is packaged into the MVs. DEX (green fluorescent) was clearly shown to be encapsulated by MVs (Figure 2E1). Moreover, we incubated Fluorescein-DEX packaging MVs with mice glomerular endothelial cells (GECs). It was found that the green MVs were efficiently taken up by GECs and the drug could enter into the nucleus (Figure 2E2). Together, these findings demonstrated that RAW macrophages indeed generated DEX-packaging MVs (named as MV-DEX).

### DEX-packaging MVs efficiently target inflamed kidney

**Figure 3:**
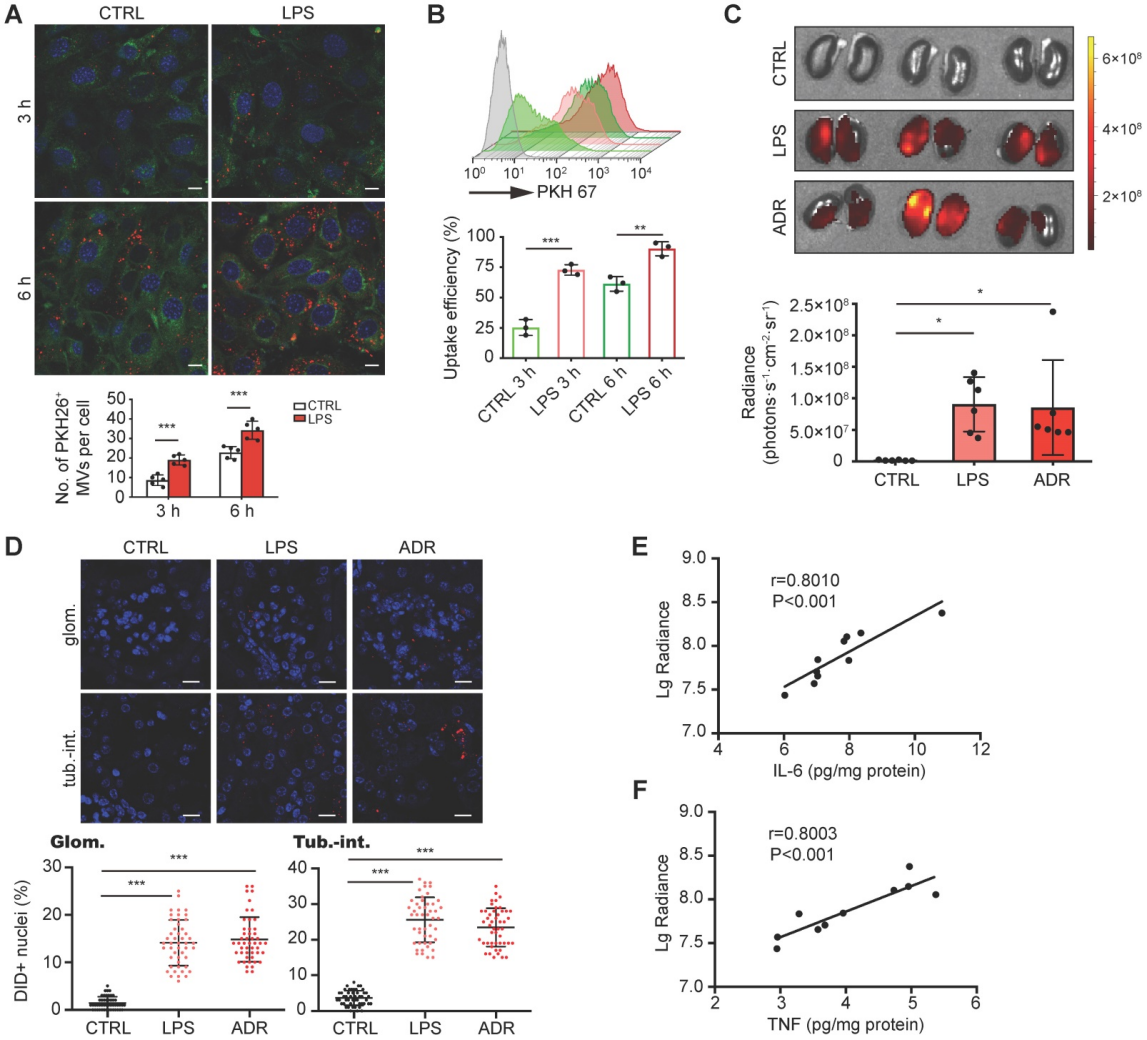
** DEX-packaging MVs efficiently target inflamed kidney.** (**A, B**) GECs took up MV-DEX. PKH 26 or PKH 67-labeled MVs (5×10^7^) were incubated with GECs for 3 or 6 h, respectively, and then PKH 26-positive (red) cells were observed under confocal microscope (**A**) (scale bar, 10 μm), while PKH 67-positive cells were analyzed by flow cytometry (**B**). Compared with control cells, LPS-treated cells took up more MV-DEX. (**C, D**) For *in vivo* distribution, mice were injected intravenously with DID-labeled MVs (1×10^10^). Imaging of the kidneys for detection of DID-labeled MVs 24 h after injection (**C**). Representative micrographs and quantification of DID-labeled MVs in kidney sections (**D**) (scale bar, 25 μm). MV-DEX showed a great increase in accumulation into inflamed kidney tissue at 24h after injection. Data are presented as mean ± SD, * p<0.05, ** p<0.01, *** p<0.001, two-tailed t-test (A, B), one-way ANOVA (C, D). (**E, F**) Positive correlation between the renal average radiance of DID and renal IL-6 (**E**) or TNF (**F**) in LPS-treated mice. n=10 kidneys. The data were compared using the Spearmen correlation coefficient.

To characterize the targeting capacity of MV- DEX, we first examined the uptake of MV-DEX by GECs *in vitro.* PKH 26-labeled MVs were incubated with GECs activated or not-activated by LPS stimulation. It was found that MV-DEX could be internalized into GECs in a time-dependent manner (Figure 3A). Increasing levels of MV-DEX were found to be internalized in the LPS-treated cells at two time points (3 and 6 h) (Figure 3A), suggesting that MV-DEX could efficiently target inflamed cells. Consistently, ~90% of GECs treated with LPS versus ~61% of control cells were found to take up MVs at 6 h by flow cytometry (Figure 3B).

Next, we investigated whether the inflammation increases the homing of MV-DEX to the kidney. Following induction of LPS or ADR nephropathy in mice, we intravenously injected DID-labeled MV- DEX. 24 h after administration, ex vivo imaging of the organs (heart, lung, liver, spleen and kidney) was performed (Figure S2A). Compared to mice without injury, significantly higher renal radiance signals were observed in LPS- or ADR-treated mice (Figure 3C). We also tracked the fluorescence levels in each tissue section (Figure S2B), which corroborated our *in vivo* findings. Specifically, robust amounts of MV- DEX accumulated in the glomeruli and tubulointerstitium of LPS- or ADR-treated mice whereas very few were detected in control mice (Figure 3D), and MVs were detectable within the endothelial cells, podocytes, tubular cells and macrophages (Figure S2C). Furthermore, the extent of renal fluorescence was positively correlated with renal inflammation as quantitated by IL-6 or TNF protein expression (Figures 3E and 3F). These findings suggested that MV-DEX exhibited preferential targeting of inflamed kidney both *in vitro* and *in vivo*.

### LFA-1 and VAL-4 guide the homing of MV-DEX to inflamed kidney

**Figure 4:**
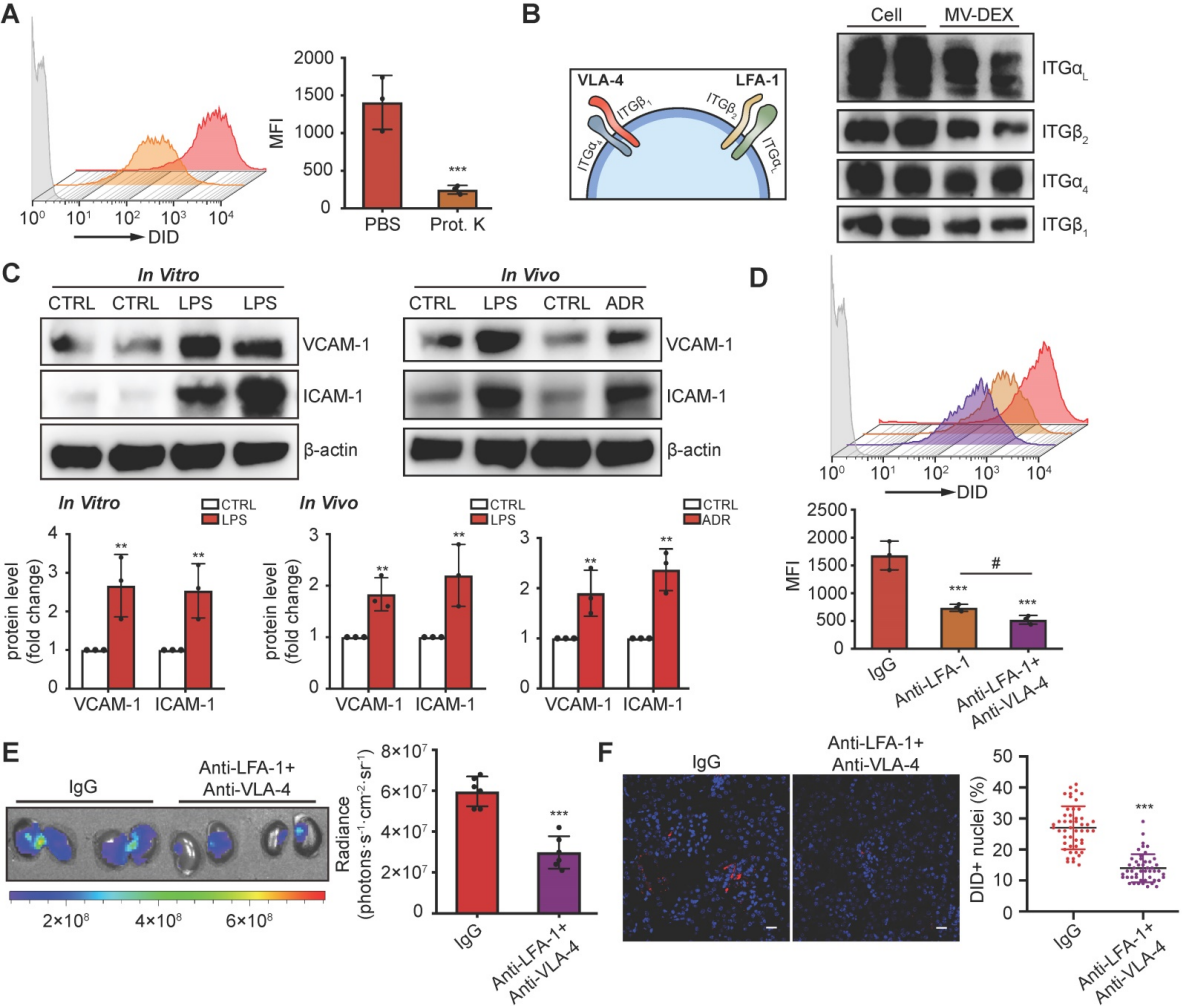
** LFA-1 and VAL-4 guide the homing of MV-DEX to inflamed kidney.** (**A**) Uptake of MV-DEX in LPS-induced GECs. Cellular uptake was determined by flow cytometry after 6 h incubation with DID-labeled MVs (5×10^7^) pre-treated with or without proteinase K. Loss of surface proteins on MV-DEX significantly reduced their entry into inflamed GECs. (**B**) Western blot analysis showed ITGα_L_, ITGβ_2_, ITGα_4_ and ITGβ_1_ were robustly expressed on MV-DEX. (**C**) Western blot analysis showed VCAM-1 and ICAM-1 were significantly increased in LPS-treated GECs or kidneys from LPS-treated or ADR-treated mice. (**D**) Effect of LFA-1 and VLA-4 blocking on cellular uptake of MV-DEX in LPS-induced GECs. Cellular uptake was determined by flow cytometry after 6 h incubation with DID-labeled MVs (5×10^7^) pre-incubated with ITGβ_2_ blocking antibody or VLA-4 blocking antibody. The uptake of MV-DEX was reduced 70% by inhibition of ITGβ_2_ and VLA-4. (**E, F**) Effect of LFA-1 and VLA-4 blocking on biodistribution of MV-DEX in LPS-treated mice. Mice were injected intravenously with DID-labeled MVs (1×10^10^) pre-incubated with ITGβ_2_ blocking antibody and VLA-4 blocking antibody. Imaging of the kidneys for detection of DID-labeled MVs 24 h after injection (**E**). Representative micrographs and quantification of DID-labeled MVs in kidney sections (**F**) (scale bar, 25 μm). MV-DEX showed a great decrease in accumulation into kidney tissue. Data are presented as mean ± SD, * p<0.05, ** p<0.01, *** p<0.001, # p<0.05, two-tailed t-test (A, C, E, F), one-way ANOVA (D).

The surface receptors of EVs allow their targeting and capture by recipient cells. Loss of surface proteins on MV-DEX with proteinase K treatment significantly reduced their entry into inflamed GECs (Figure 4A), indicating that proteins on MV-DEX have a critical role in targeting inflamed endothelium. Monocytes are known to elicit homing affinities to inflamed tissues through integrin-mediated adhesive interactions, such as lymphocyte function-associated antigen 1 (LFA-1; α_L_β_2_-integrin)/ICAM-1 and very late antigen 4 (VLA-4; α_4_β_1_-integrin)/VCAM-1 interaction 33,34. We investigated whether these molecules were maintained on MV-DEX using mass spectrometry, and found distinct integrin expression profiles correlated with leukocyte adhesion (Supplementary Table 1). Further, the expression of integrin α_L_ (ITGα_L_), ITGβ_2_, ITGα_4_ and ITGβ_1_ on MV-DEX was validated by western blot (Figure 4B). Therefore, we proposed that LFA-1 and VLA-4 guide the homing of MV-DEX to inflamed kidney, in which ICAM-1 and VCAM-1 were indeed overexpressed during injury (Figure 4C). To test this idea, we incubated MV-DEX with blocking antibodies against ITGβ_2_ (LFA-1) and VLA-4 or with the corresponding isotype control, and found that anti-ITGβ_2_ antibodies or its combination with anti-VLA-4 antibodies significantly inhibited MV uptake in inflamed GECs (Figure 4D). In addition, the accumulation of MV-DEX treated by a panel of anti- ITGβ_2_ antibodies and anti-VLA-4 antibodies was also similarly decreased in kidneys of LPS-treated mice (Figures 4E and 4F), indicating LFA-1 and VLA-4 present on MV-DEX are responsible for their homing to inflamed kidney. Notably, considering that renal inflammation is induced in a wide range of injuries 22,23,35-37, macrophage-derived MVs could be utilized as drug carriers to target inflammation in most renal diseases.

### Enhanced anti-inflammatory efficacy of MV-DEX *in vitro*

Glucocorticoids exert anti-inflammatory activities by forming glucocorticoid-glucocorticoid receptor (GR) complexes, which can upregulate the expression of anti-inflammatory genes (transactivation) or repress the nuclear translocation of proinflammatory transcription factors (transrepression), such as NF-κB 1,2,38. Thus, we detected the expression of proinflammatory cytokines and the activation of NF-κB signaling to assess the anti-inflammatory capacity of MV-DEX in LPS-treated GECs. It was found that MV- DEX dose-dependently inhibited LPS-induced mRNA expression of TNF, IL-6, IL-β and CCL-2 (Figure S4A), while drug-free MVs derived from normal RAW cells showed no significant therapeutic effects (Figure S3A). Moreover, we filtered the pellet obtained by centrifugation with filters of different pore sizes, and found that reducing MV-DEX from the pellet by passaging it through a 0.1-μm filter abrogated their ability to repress inflammation (Figure S4B), indicating that MV-DEX, rather than other soluble factors contaminating the pellet, was responsible for the anti-inflammatory efficacy. With regard to other cells (including podocytes and tubular cells), MV-DEX could also inhibit LPS-induced mRNA expression of proinflammatory cytokines *in vitro* (Figure S4C). These findings suggested that the MV-DEX was indeed capable of alleviating inflammation *in vitro*.

**Figure 5:**
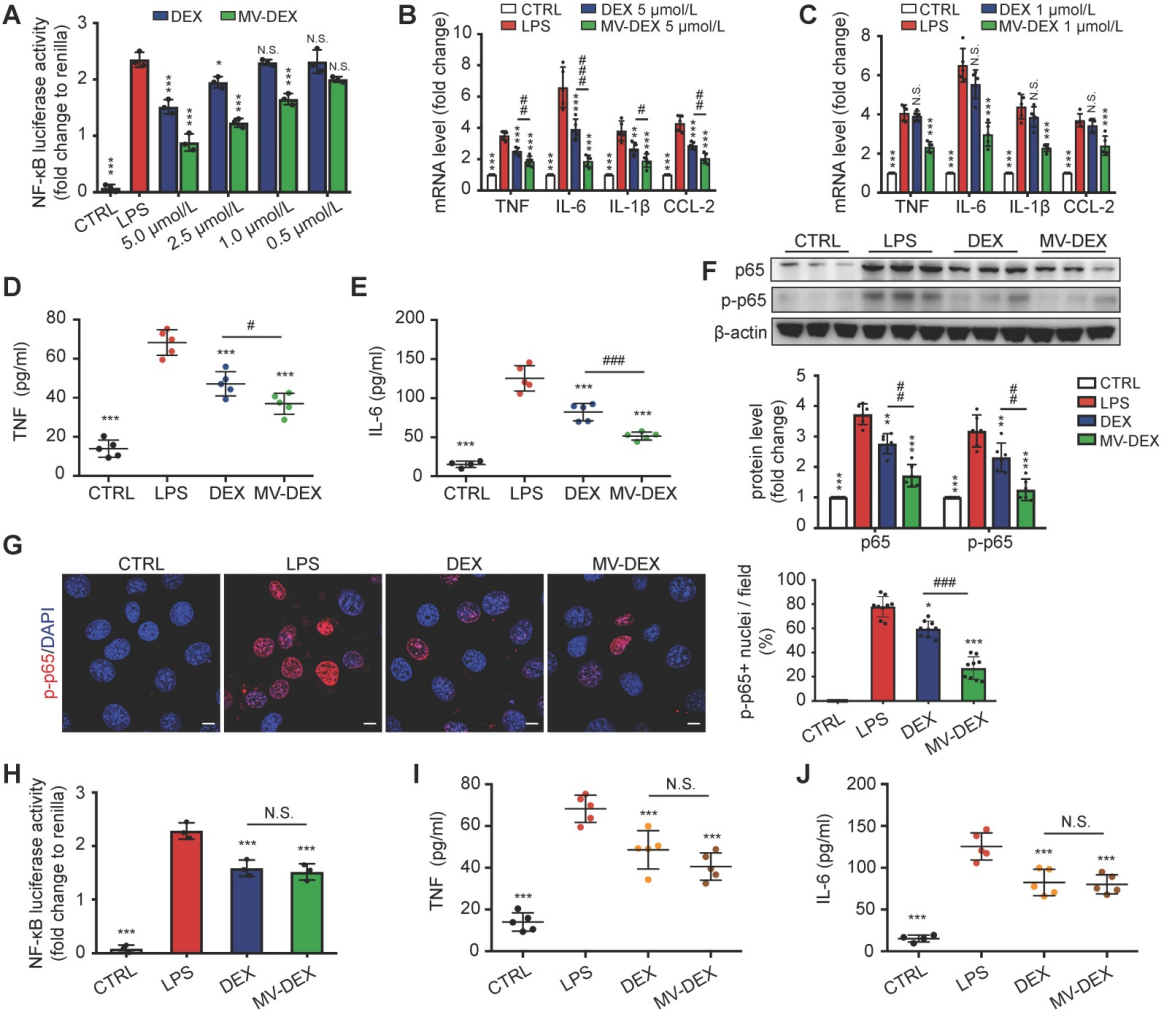
** Enhanced anti-inflammatory efficacy of MV-DEX *in vitro*.** GECs were stimulated with LPS and treated with the indicated concentrations of MV-DEX or free DEX for 12 h. (**A**) Effects of MV-DEX or free DEX on NF-κB luciferase reporter activity in LPS-induced GECs. Compared with free DEX, MV-DEX showed a 5-fold increase in inhibiting NF-κB activity. (**B, C**) Real-time PCR analysis of inflammatory cytokines (TNF, IL-6, IL-β and CCL-2) in GECs. TNF (**D**) and IL-6 (**E**) levels in the supernatants were detected by ELISA. (**F**) The expression of p65 and p-p65 were analyzed by western blot. (**G**) Immunofluorescent staining against p-p65 was observed under confocal microscope (scale bar, 10 μm). Both MV-DEX and free DEX exhibited anti-inflammatory efficacy, but MV-DEX was much more superior. (**H-J**) MV-DEX and free DEX were stored at 37 ℃ for 10 days to destroy most MVs, whereas the drug concentration between two groups remained the same (with 5 μmol/L DEX). NF-κB luciferase reporter activity was shown as fold change to renilla luciferase activity (**H**). ELISA detection of TNF (**I**) and IL-6 (**J**) levels in supernatants. Following MV destruction, the MV-DEX group no longer showed a superior inhibition of inflammation compared with free DEX. Data are presented as mean ± SD, ** p<0.01, *** p<0.001 vs. LPS-treated group, # p<0.05, ## p<0.01, ### p<0.001, N.S., not significant, one-way ANOVA.

Next, we compared the anti-inflammatory efficacy between free DEX and MV-DEX with equimolar doses. MV-DEX reduced NF-κB luciferase reporter activity to a greater magnitude than free DEX therapy (Figure 5A), as well as the expression of NF-κB-mediated proinflammatory genes (Figures 5B and 5C). Notably, MV-DEX showed a comparable treatment efficacy only with one-fifth of the doses of DEX, suggesting that MV-directed delivery of DEX could greatly reduce the therapeutic doses and mitigate side effects accordingly. Moreover, inhibition of TNF and IL-6 in the supernatants (Figures 5D and 5E) and repression of NF-κB p65, p-p65 in the GECs (Figures 5F and 5G) were also enhanced in MV-DEX group. Collectively, MV-DEX exhibited superior anti-inflammatory efficacy in comparison with free DEX *in vitro*.

To determine whether MV-mediated drug delivery was responsible for this enhancement, we stored the MV-DEX and free DEX (5 μmol/L) at 37 ℃ for 10 days to destroy most MVs (Figure S1C), whereas the drug concentration between two groups remained the same. It was found that, following MV destruction, the MV-DEX group no longer showed a superior inhibition of NF-κB luciferase reporter activity as well as TNF and IL-6 (Figures 5H-5J). Moreover, we also found that MV destruction made the therapeutic efficacy of MV-DEX no different from that of free DEX in LPS-treated mice (Figure S5), confirming that delivery of DEX in MVs was more effective than direct treatment with the drug.

### Enhanced renoprotective and anti-inflammatory efficacy of MV-DEX in LPS-treated mice

**Figure 6:**
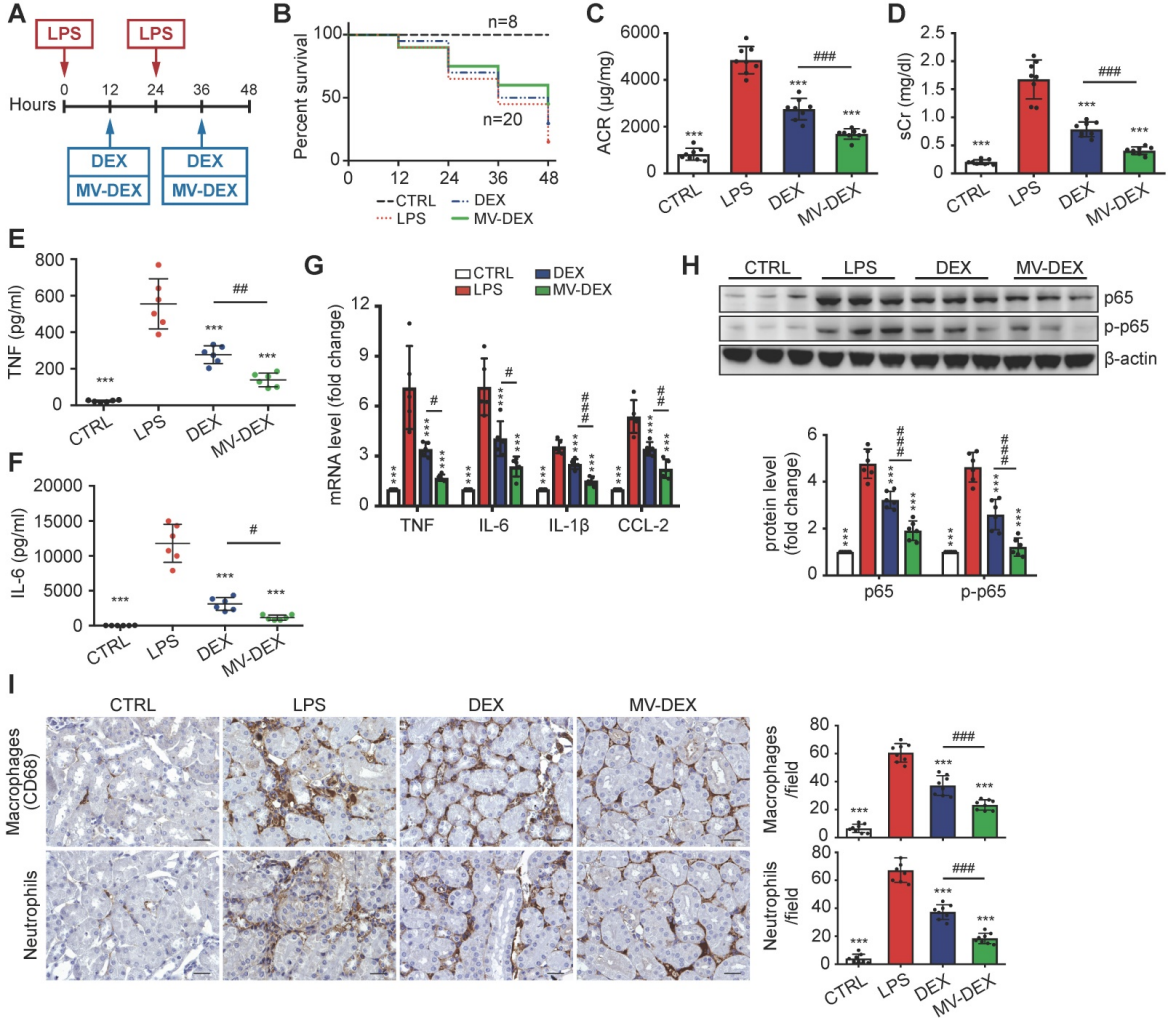
** Enhanced renoprotective and anti-inflammatory efficacy of MV-DEX in LPS-treated mice.** (**A**) Schematic diagram of the experimental design. In brief, mice were concurrently treated with MV-DEX or free DEX or vehicle after LPS treatment, and were sacrificed 48 h after disease induction. (**B**) Survival curve reveled enhanced survival in MV-DEX-treated mice with 45% survival compared to 30% in the free DEX-treated mice. n=8 mice (CTRL); n=20 mice (DEX, MV-DEX). (**C**) Albuminuria (urine albumin-to-creatinine ratio) and serum creatinine (**D**) were reduced to a much greater extent after MV-DEX treatment than with free DEX treatment. n=8 mice per group. ELISA detection of TNF (**E**) and IL-6 (**F**) levels in mice's serum. Real-time PCR analysis of cytokine (TNF, IL-6, IL-β and CCL-2) mRNA expression levels (**G**) and Western blot analysis of p65 and p-p65 (**H**) in renal cortex tissue lysates. (**I**) Immunostaining and quantification of macrophages and neutrophils in the tubulointerstitium (scale bar, 25 μm). MV-DEX had a more potent ability to alleviate renal inflammation. n=6 mice per group. Data are presented as mean ± SD, *** p<0.001 vs. LPS-treated mice, # p<0.05, ## p<0.01, ### p<0.001, one-way ANOVA.

Next, we used the LPS model to address whether MV-DEX could be used for anti-inflammatory therapy *in vivo* (Figure 6A). The treatment protocol is described in detail in the Methods. MV-DEX-treated mice showed enhanced survival, with 45% survival compared to 30% in the free DEX-treated mice (Figure 6B). Additionally, MV-DEX significantly reduced serum creatinine and albuminuria compared to free DEX (Figures 6C and 6D), whereas drug-free MVs derived from RAW cells had no effect (Figures S3B and S3C). DEX has been proven to protect podocytes from LPS injury 27,28. Here, we confirmed that MV-DEX improved the therapeutic efficacy of DEX on podocytes based on immunofluorescent analysis of WT-1 and nephrin, two specific biomarkers of podocyte (Figures S6). Collectively, these data demonstrate excellent therapeutic efficacy of MV-DEX against proteinuria and glomerular lesions in the LPS model.

Further, TNF and IL-6 levels in the serum (Figures 6E and 6F), mRNA expression of TNF, IL-6, IL-β and CCL-2 in the kidney (Figure 6G), and protein expression of NF-κB p65 and p-p65 (Figure 6H, Figure S7A) were substantially decreased in MV-DEX- treated mice. These inflammatory mediators were also blunted with free DEX, however, to a much lesser extent than with MV-DEX. In addition, the immunostaining against CD68 and neutrophil showed that MV-DEX markedly inhibited the interstitial infiltration of macrophages and neutrophils (Figure 6I). Also, expression of CCL-2, TNF and ICAM-1 in the glomeruli and tubule were downregulated by MV- DEX (Figure S7B-S7D), and the efficacy of MV-DEX was greater than that of free DEX. Above all, these data suggested that MV-DEX had a more potent ability to alleviate renal inflammation.

### Enhanced renoprotective and anti-inflammatory efficacy of MV-DEX in ADR-treated mice

**Figure 7:**
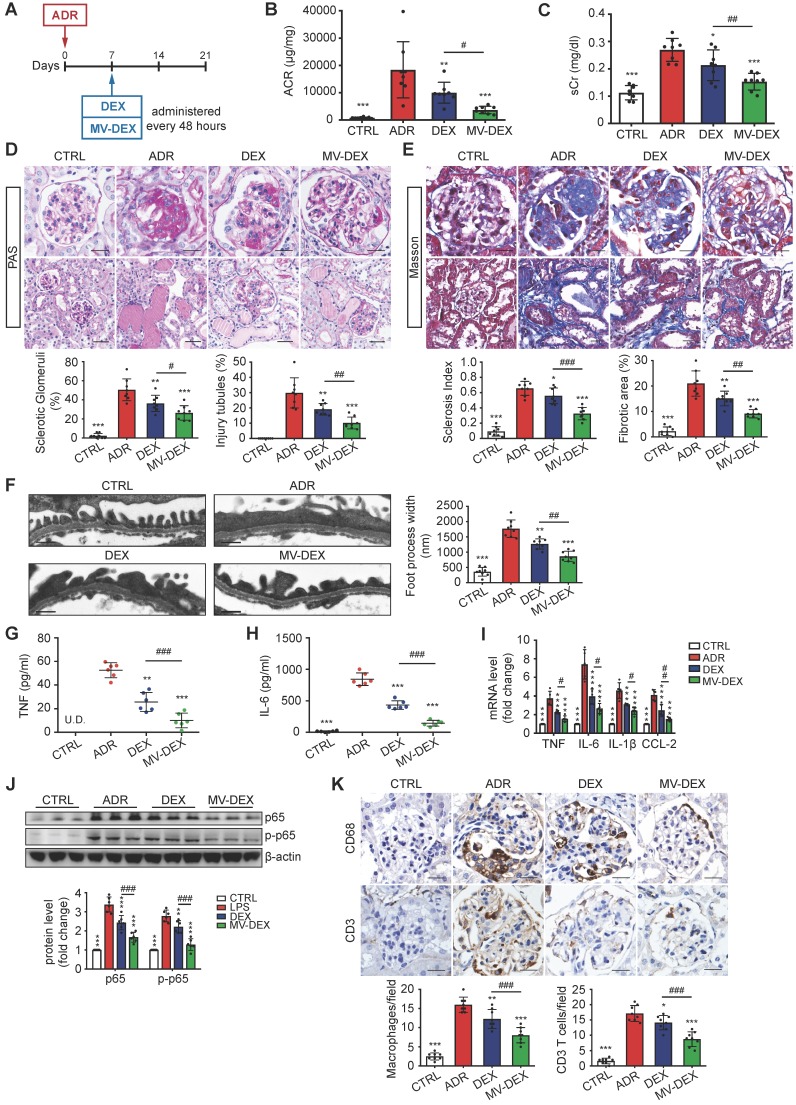
** Enhanced renoprotective and anti-inflammatory efficacy of MV-DEX in ADR-treated mice.** (**A**) Schematic diagram of the experimental design. In brief, 7 days after injection of ADR, mice were treated with free DEX or MV-DEX (0.5 mg/kg every 48 hours) until 3 weeks post-ADR treatment. (**B**) Albuminuria (urine albumin-to-creatinine ratio) and (**C**) serum creatinine were significantly reduced in MV-DEX-treated mice compared to free DEX-treated mice. (**D**) Representative images of PAS staining. Top, glomerular morphology; Bottom, tubulointerstitial morphology. (**E**) Representative images of Masson trichrome staining. Top, glomerulosclerosis; Bottom, tubulointerstitial fibrosis. scale bars, 25 μm. n=8 mice per group. (**F**) Electron microscopy was performed to assess ultrastructural changes in podocyte morphology (scale bar, 200 nm). n=4 mice per group. All the histological analysis showed a better renoprotection effect of MV-DEX. (**G, H**) ELISA detection of TNF and IL-6 in mice's serum. Real-time PCR analysis of inflammatory cytokine mRNA expression levels (**I**) and western blot analysis of p65 and p-p65 (**J**) in renal cortex tissue lysates. (**K**) Immunostaining and quantification of macrophages and T cells in the glomeruli (scale bar, 25 μm). Compared to free DEX treatment, MV-DEX showed more potent in reducing renal inflammation in ADR-induced nephropathy. n=6 mice per group. Data are presented as mean ± SD, * p<0.05, ** p<0.01, *** p<0.001 vs. ADR-treated mice, # p<0.05, ## p<0.01, ### p<0.001, one-way ANOVA.

Next, we investigated the potential therapeutic efficacy of MV-DEX in a murine model of ADR- induced nephropathy. At day 7 post-ADR treatment, MV-DEX or free DEX was initiated intravenously and continued every 48 h for 2 weeks (Figure 7A). The detailed description of the protocol is provided in the Methods. In contrast to free DEX, MV-DEX treatment caused a more obvious reduction in albuminuria and serum creatinine (Figures 7B and 7C). ADR nephrosis was characterized by severe lesions in both glomerular and tubular compartments. As expected, segmental glomerulosclerosis (Figures 7D and 7E), podocyte injury (Figure 7F, Figures S8) and interstitial fibrosis (Figure 7E) each were significantly ameliorated in mice received MV-DEX treatment compared with that received free DEX treatment. While no therapeutic effects were observed in drug-free MVs- treated mice (Figures S3D-S3F). Notably, our data supported the more beneficial effects of MV-DEX to attenuate proteinuria, glomerular lesions, and tubuleinterstitial injury.

Moreover, we examined the efficacy of MV-DEX treatment in reducing renal inflammation. The induction of ADR nephrosis significantly unregulated the protein levels of TNF and IL-6 in the serum (Figures 7G and 7H), mRNA expression of TNF, IL-6, IL-β and CCL-2 in the kidney (Figure 7I), and protein expression of NF-κB p65 and p-p65 (Figure 7J, Figure S9A), which were downregulated by MV-DEX to a greater extent than free DEX treatment. In addition, we showed that glomerular infiltration of macrophages (CD68) and CD3 T cells (Figure 7K), protein expression of CCL-2, TNF and ICAM-1 in the glomeruli and tubule (Figures S9B-S9D) were also significantly diminished in MV-DEX-treated mice compared with DEX-treated mice. Together, MV-DEX was proven more potent in reducing expression of inflammatory mediators in ADR-induced nephropathy, leading to superior anti-inflammatory effects.

### GR in DEX-packaging MVs facilitate the anti-inflammatory efficacy

**Figure 8:**
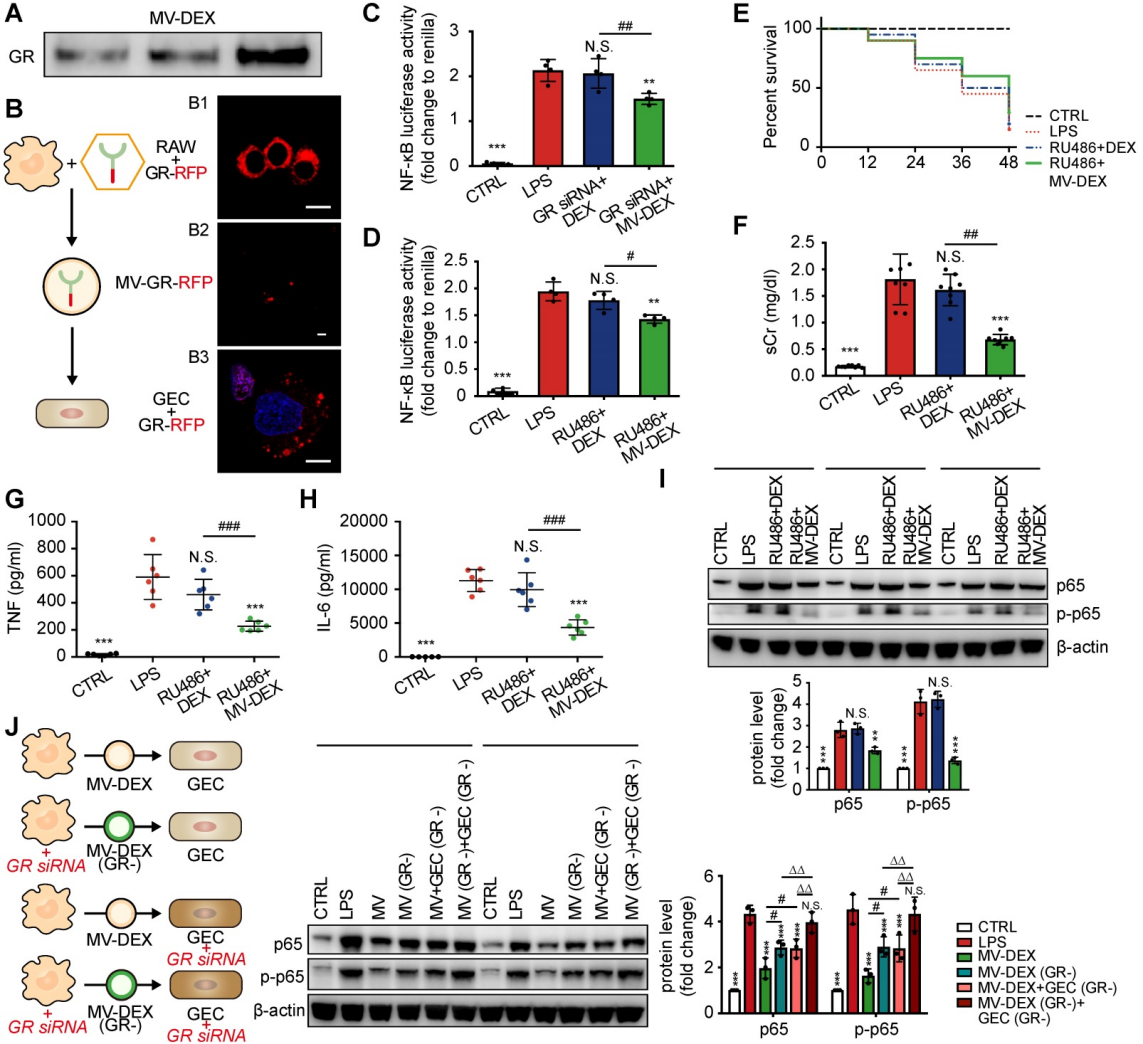
** Glucocorticoid receptor (GR) in DEX-packaging MVs facilitate the anti-inflammatory efficacy.** (**A**) GR in MV-DEX was detected by Western blot. n=3. (**B**) RAW cells were transfected with RFP-tagged GR (B1) to trace the location of the receptor. MVs were isolated and RFP-tagged GR (red fluorescent) was observed in MVs (B2) (scale bar, 1 μm). GECs were incubated with GR-RFP packaging MVs for 12 h and then observed under confocal microscopy (B3) (scale bar, 10 μm). (**C, D**) GECs were transfected with siRNA or treated with RU486 to downregulate GR expression. NF-κB luciferase reporter activity revealed MV-DEX significantly reduced NF-κB activity, while the ability of free DEX was abrogated. n=4. (**E-I**) After intraperitoneal injection of RU486 for 7 days, LPS-induced nephropathy model and treatment protocol were built as previously described. (**E**) Survival curve showed mice received MV-DEX treatment had lower mortality compared to mice received free DEX treatment. n=8 mice (CTRL); n=20 mice (RU486+DEX, RU486+MV-DEX). Serum creatinine (**F**), TNF (**G**) and IL-6 (**H**) levels were also reduced to a much greater extent after MV-DEX treatment than with free DEX treatment. n=6 mice per group. (**I**) Western blot analysis of p65 and p-p65 in renal cortex tissue lysates showed MV-DEX was more superior in reducing NF-κB activity. n=3. (**J**) RAW cells were transfected with siRNA to reduce GR expression in the MV-DEX, also GECs were transfected to reduce endogenous GR expression. The experimental groups were as follows: GECs treated by MV-DEX with normal GR (MV-DEX group), GECs treated by MV-DEX with low GR (MV-DEX(GR-) group), GECs with low GR treated by MV-DEX with normal GR (MV-DEX+GEC(GR-) group), GECs with low GR treated by MV-DEX with low GR (MV-DEX(-)+GEC(GR-) group). Expression levels of p65 and p-p65 were detected by Western blot. Reducing GR expression in the MV-DEX or GECs both abated the anti-inflammatory efficacy of MV-DEX. n=3. Data are presented as mean ± SD, * p<0.05, ** p<0.01, *** p<0.001 vs. LPS-treated group, # p<0.05, ## p<0.01, ### p<0.001, N.S., not significant, one-way ANOVA.

It has long been known that glucocorticoids function through activation of the glucocorticoid receptor (GR) (encoded by NR3C1), which is expressed in many types of renal cells, including endothelial cells, mesangial cells, podocytes and tubular cells 39. Here, we found that MV-DEX treatment caused a more obvious enhancement in expression of GR both in cultured GECs and *in vivo* kidney compared to free DEX treatment (Figures S10). As cell sensitivity to glucocorticoids is associated with cellular levels of the GR, we hypothesized that MV-DEX could package and deliver GR to renal cells, thereby, increasing cellular levels of the receptor and improving the anti-inflammatory efficacy. Indeed, GR was detected in MV-DEX by Western blot (Figure 8A). To further ascertain that GR can be delivered by the MV-DEX, red fluorescent protein (RFP)-tagged GR was expressed in RAW cells (Figure 8B1) and the MVs were isolated (Figure 8B2) and incubated with GECs. As expected, the RFP-tagged GR (red fluorescent) was shown in the cytoplasm of GECs and could translocate to the nucleus (Figure 8B3). Our findings illustrated that cytoplasm protein GR in macrophages could be delivered into renal cells by MVs.

Next, we downregulated the expression of GR in GECs by siRNA (Figures S11A) or pharmacologic GR antagonist RU486 40 (Figures S11B), respectively. It was found that MV-DEX administration still reduced NF-κB luciferase reporter activity despite the low expression of GR, while the anti-inflammatory ability of DEX was abrogated (Figures 8C and 8D). In addition, mice were treated daily by intraperitoneal injections of RU486 for 7 days to decrease GR expression in kidneys (Figures S11C), and then LPS-induced nephropathy and treatment protocol were conducted as previously described. Mice received MV-DEX treatment showed lower mortality compared to mice received DEX treatment (Figure 8E). Also, MV-DEX treatment reduced serum creatinine (Figure 8F), TNF and IL-6 levels (Figures 8G and 8H), and inhibited NF-κB p65 and p-p65 expression in the kidney (Figure 8I), while no significant therapeutic effects were observed in free DEX-treated group owing to low expression of GR in the kidney. Collectively, it was reasonably to assume that GR in the MVs could compensate for the loss of GR in kidneys.

Further, we investigated the effect of GR in the MVs and endogenous GR in the GECs on the anti-inflammatory efficacy of MV-DEX. RAW cells were transfected with siRNA to reduce GR expression in the MV-DEX (Figures S11D), and GECs were transfected to reduce endogenous GR expression (Figures S11A). It was found that reducing GR expression in the MVs or GECs both abated the anti-inflammatory efficacy of MV-DEX (Figure 8J). Additionally, when GECs had low endogenous GR level, the loss of GR in the MVs abrogated its anti-inflammatory capacity (Figure 8J). Our results demonstrated that GR in both MVs and GECs were responsible for the therapeutic efficacy of MV-DEX, and what's more, GR in the MVs could increase cellular levels of the receptor and facilitate the anti-inflammatory efficacy.

Many factors can affect the cellular levels of GR, thereby diminishing the efficacy of GR-dependent therapeutics. For example, hypoxia reduces GR function and its ligand binding ability in the fetal heart 41. The NLRP3/caspase-1 axis, which propagates renal inflammation 42-44, cleaves cellular GR and diminishes cell sensitivity to glucocorticoids 45. Moreover, many studies point to glucocorticoid- mediated GR downregulation as a primary mechanism for acquired resistance 46,47. Interestingly, MV-DEX could package and deliver GR to renal cells, thereby, increasing cellular levels of the receptor and improving cell sensitivity to DEX. We believe MV-DEX can be further developed into a viable strategy to improve the susceptibility or resistance to glucocorticoids.

### MV-DEX therapy ameliorates the adverse effects of DEX in ADR-treated mice

**Figure 9:**
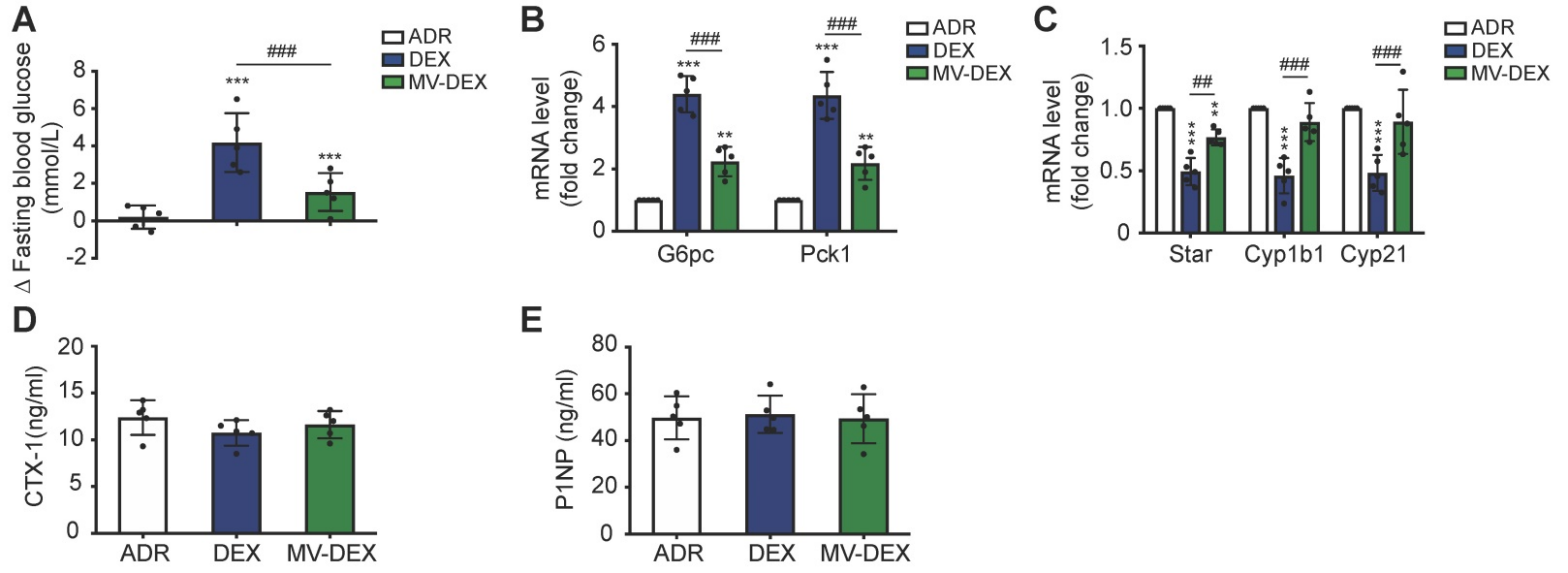
** MV-DEX therapy ameliorates the adverse effects of DEX in ADR-treated mice.** (**A**) Fasting (6 h) blood glucose (change (Δ) between 14 days of treatment) measured in ADR-induced mice treated with vehicle, free DEX or MV-DEX. (**B**) Real-time PCR analysis of gluconeogenic genes in livers. (**C**) Real-time PCR analysis of steroidogenic genes in adrenals. (**D**)(**E**) ELISA detection of plasma markers of bone metabolism. n=5 mice per group. Data are presented as mean ± SD, ** p<0.01, *** p<0.001 vs. ADR-treated mice, ## p<0.01, ### p<0.001, one-way ANOVA.

Prolonged glucocorticoid treatment can result in multiple deleterious adverse effects, such as hyperglycemia, infection, osteoporosis and suppression of the hypothalamic-pituitary-adrenal (HPA) axis 1,2. To determine if MV-mediated delivery circumvents many of the side effects, we investigated the impacts of MV-DEX on liver, adrenals and bone. Compared to mice treated with free DEX, MV-DEX treatment significantly lowered fasted blood glucose levels (Figure 9A) and gluconeogenic markers G6pc and Pck1 expression in liver (Figure 8B), which might be because MV-mediated delivery reduced the drug concentration in the liver (Figures S13). DEX mono- therapy suppressed the expression of steroidogenic genes in adrenals (Figure 9C), while MV-DEX treatment had limited impacts of these transcriptional factors (Figure 9C). Further, none of the treatments induced changes in plasma markers of bone metabolism: C-terminal telopeptides of type I collagen (CTX-I) and N-terminal propeptide of type Ⅰ collagen (PⅠNP) (Figures 9D and 9E). In addition, no significant immune response or antibody production against antigens on MVs were found after MV-DEX therapy (Figures S14). Collectively, these findings revealed that MVs enable safe and effective delivery of DEX for the treatment of kidney diseases.

### Macrophage MVs facilitate the entry of DEX into the cells

The poor solubility of synthetic dexamethasone significantly decreases drug bioavailability with consequent diminution of their therapeutic efficacy 48. EV encapsulation is able to modify the bioavailability of the native drug. For instance, exosomal formulation enhances the bioavailability of celastrol and curcumin, leading to better therapeutic responses 15, 49. In our study, MVs could facilitate the entry of DEX into GECs (Figures S15A-S15C) and thereby increase cellular drug concentrations (Figure S15D), which is one of the underlying mechanisms for enhanced ant-inflammatory efficacy of MV-DEX.

## Conclusion

In our study, macrophage-derived MVs provided a feasible means to deliver DEX to the inflamed kidney, exerting superior renoprotection with enhanced anti-inflammatory and anti-fibrotic efficacy in comparison with free DEX. LFA-1 and VLA-4 present on MV-DEX were responsible for their homing to the inflamed kidney. Notably, MV-DEX therapy reduced the deleterious side effects indicative of systemic glucocorticoid action. We believe MV-DEX can be further developed into an effective and safe medical treatment for multiple renal disorders, such as IgA nephropathy, focal segmental glomerulosclerosis, minimal change disease, lupus nephritis, and membranous nephropathy, in which glucocorticoids have been extensively used as first-line therapy 1,2. In addition, adjunct proteins in the MV-DEX such as glucocorticoid receptor could compensate for the loss of the receptor in kidneys and improve susceptibility to glucocorticoids, which should be salutary for many steroid resistant patients that with low expression or low affinity of GR 1,2. Taken together, our study will open new opportunities for glucocorticoid therapy and elucidate a novel approach for drug delivery in the treatment of kidney diseases.

## Supplementary Material

Supplementary figures and tables.

Supplementary material LCMSMS.

## Abbreviations

MVs: microvesicles
DEX: dexamethasone
MV- DEX: dexamethasone-loaded macrophage-derived microvesicles
EVs: extracellular vesicles
GR: glucocorticoid receptor
HPA: hypothalamic-pituitary -adrenal
LFA-1: lymphocyte function-associated antigen 1
VLA-4: very late antigen 4
LPS: lipoploysaccharide
ADR: adriamycin
DID: 1,1'-dioctadecyl- 3,3,3',3'-tetramethylindodicarbocyanine perchlorate
LC-MS/MS: liquid chromatography-tandem mass spectrometry
HPLC: high-performance liquid chromatography
TEM: transmission electron microscopy
PAS: Periodic Acid-Schiff
ACR: urine albumin/urine creatinine
sCr: serum creatinine
