# Employing Macrophage-Derived Microvesicle for Kidney-Targeted Delivery of Dexamethasone: An Efficient Therapeutic Strategy against Renal Inflammation and Fibrosis: Erratum

**DOI:** 10.7150/thno.78981

**Published:** 2023-01-09

**Authors:** Tao-Tao Tang, Lin-Li Lv, Bin Wang, Jing-Yuan Cao, Ye Feng, Zuo-Lin Li, Min Wu, Feng-Mei Wang, Yi Wen, Le-Ting Zhou, Hai-Feng Ni, Ping-Sheng Chen, Ning Gu, Steven D. Crowley, Bi-Cheng Liu

**Affiliations:** 1 Institute of Nephrology, Zhong Da Hospital, School of Medicine, Southeast University, Nanjing, China; 2 State Key Laboratory of Bioelectronics, Jiangsu Key Laboratory for Biomaterials and Devices, School of Biological Sciences and Medical Engineering, Southeast University, Nanjing, China; 3 Division of Nephrology, Department of Medicine, Duke University and Durham VA Medical Centers, Durham, North Carolina, United States

The authors apologize that the original version of the above article contains errors that need to be corrected. An incorrect image of neutrophil staining of the MV-DEX group in Figure 6I was used in figure assembly. The authors apologize for any inconvenience the error may have caused. Luckily the correction does not affect the conclusions of the above paper. The corrected Figure 6I appears below.

## Figures and Tables

**Figure 6:**
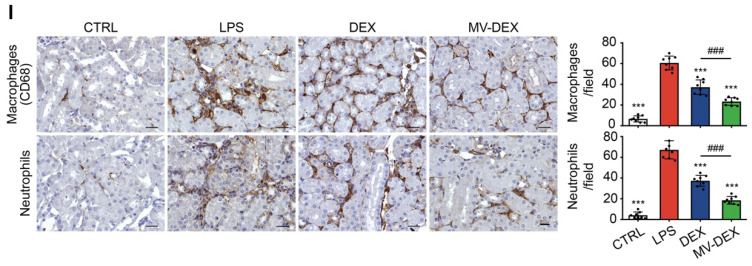
Corrected figure for original Figure 6I.

