# Supplementary material for: Employing Macrophage-Derived Microvesicle for Kidney-Targeted Delivery of Dexamethasone: An Efficient Therapeutic Strategy against Renal Inflammation and Fibrosis
Source: Theranostics. 2019 Jul 9;9(16):4740–55. doi: 10.7150/thno.33520 (PMC6643445; doi:10.7150/thno.33520)

Employing macrophage-derived microvesicle for kidney-targeted delivery of dexamethasone: An efficient therapeutic strategy against renal inflammation and fibrosis

Authors:

Tao-Tao Tang,[1] Lin-Li Lv,[1,†] Bin Wang,[1] Jing-Yuan Cao,[1] Ye Feng,[1] Zuo-Lin Li,[1] Min Wu,[1] Feng-Mei Wang,[1] Yi Wen,[1] Le-Ting Zhou,[1] Hai-Feng Ni,[1] Ping-Sheng Chen,[1] Ning Gu,[2] Steven D. Crowley,[3] Bi-Cheng Liu[1,*]

Supplementary tables and figures

Table 1. Integrin expression in MV-DEX.

Table 2. Primers used in this study.

Fig. 1. Characterization of RAW 264.7 macrophages-derived DEX-packaging MVs.

Fig. 2. Tissue distribution of DEX-packaging MVs.

Fig. 3. Drug-free MVs derived from RAW macrophages showed no therapeutic effects.

Fig. 4. The anti-inflammatory efficacy of MV-DEX in vitro.

Fig. 5. Effect of MV destruction on the anti-inflammatory efficacy of MV-DEX.

Fig. 6. MV-DEX attenuate podocyte injury in LPS-treated mice.

Fig. 7. Enhanced anti-inflammatory efficacy of MV-DEX in LPS-treated mice.

Fig. 8. MV-DEX attenuate podocyte injury in ADR-treated mice.

Fig. 9. Enhanced anti-inflammatory efficacy of MV-DEX in ADR-treated mice.

Fig. 10. MV-DEX increase the expression of GR in vivo and in vitro.

Fig. 11. Downregulation of GR in vitro and in vivo.

Fig. 12. Characterization of primary glomerular endothelial cells (GECs).

Fig. 13. HPLC analysis of the DEX concentration in liver and kidney.

Fig. 14. Immunogenicity of MV-DEX.

Fig. 15. Macrophage MVs facilitate the entry of DEX into the cells.

Supplementary Table 1 Integrin expression in MV-DEX.

Integrins	MV-DEX		
	**Sample 1**	**Sample 2**	**Sample 3**
α_4	+	+	+
α_5	+	+	+
α_D	+	-	-
α_L	+	-	-
α_M	+	+	+
α_V	-	+	+
β_1	+	+	+
β_2	+	+	+
β_5	+	+	+

Proteomic analysis of integrins in MV-DEX; '+' indicates positive for integrin expression and '-' indicates undetected by mass spectrometry

Supplementary Table 2 Primers used in this study.

Gene	Forward	Reverse
**β-actin-HOMO**	CTACCTCATGAAGATCCTCACCGA	TTCTCCTTAATGTCACGCACGATT
**CCL-2-HOMO**	CTTGGGTTGTGGAGTGAGTGT	AGCAGAAGTGGGTTCAGGATT
**TNF-α-HOMO**	CGAAGTGGTGGTCTTGTTGCT	CCCGACTATCTCGACTTTGCC
**IL-1β-HOMO**	GTGGTGGTCGGAGATTCGTAG	GAAATGATGGCTTATTACAGTGGC
**IL-6-HOMO**	GCTCTGGCTTGTTCCTCACTA	AATCATCACTGGTCTTTTGGAG
**β-actin-MUS**	GGGAAATCGTGCGTGAC	AGGCTGGAAAAGAGCCT
**CCL-2-MUS**	TTGAGGTGGTTGTGGAAAAGG	GTGCTGACCCCAAGAAGGAAT
**TNF-α-MUS**	AGACAGAGGCAACCTGACCAC	GCACCACCATCAAGGACTCAA
**IL-1β-MUS**	GGTAAGTGGTTGCCCATCAGA	GTCGCTCAGGGTCACAAGAAA
**IL-6-MUS**	GTCACCAGCATCAGTCCCAAG	CCCACCAAGAACGATAGTCAA
**Star-MUS**	AAGAGCTCAACTGGAGAGCAC	TACTTAGCACTTCGTCCCCGT
**Cyp1b1-MUS**	TCCTCTCTGCCGAAAAGAAA	ACAACCTGGTCCAACTCAGC
**Cyp21-MUS**	AGACCCTTCACGACTGTGTC	AGACCCTTCACGACTGTGTC

Supplementary Figure 1

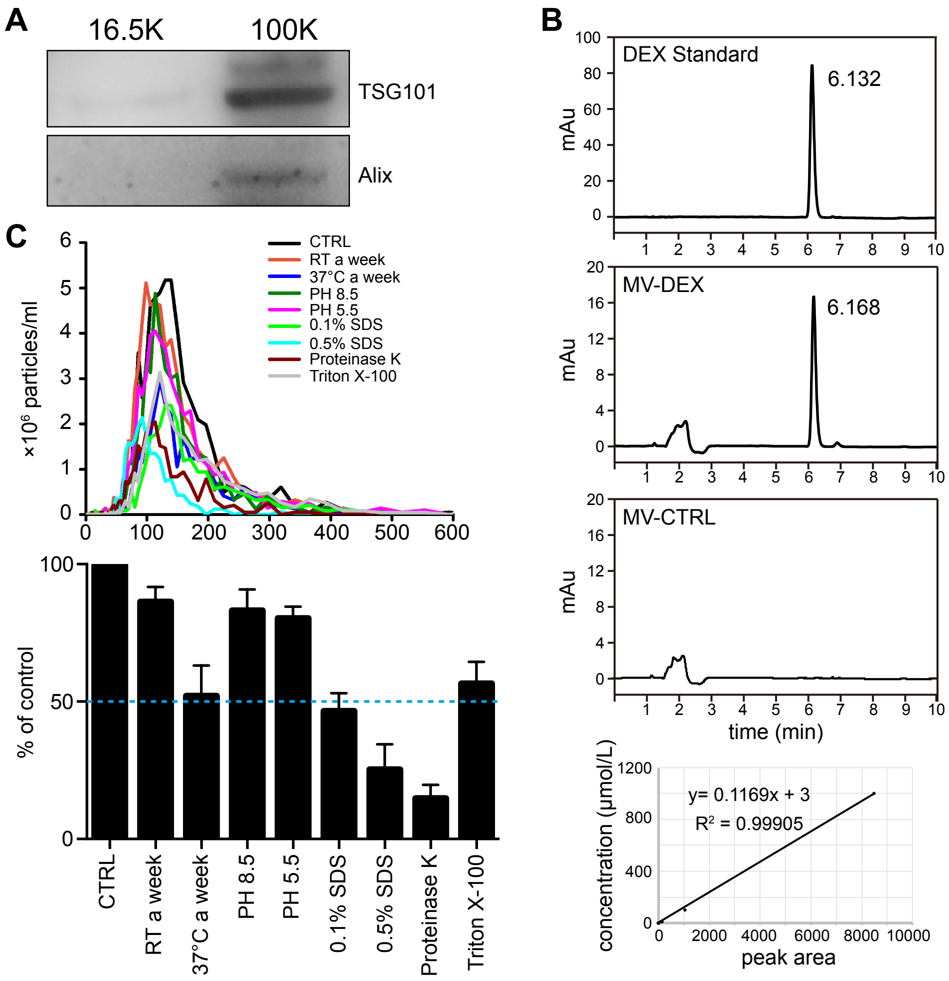

Fig.1 Characterization of RAW 264.7 macrophages-derived DEX-packaging MVs. (**A**) RAW cells were incubated with DEX and then supernatants were subjected to differential centrifugation. Brief protocol: centrifugation at 2000 × g for 20 min to remove cell debris and apoptotic bodies, followed by centrifugation at 16,500 × g for 30 min (16.5K pellet), followed by 100,000 × g for 60 min (100K pellet). Exosomal markers (TSG101 and Alix) were detected in 100K pellet, but not detected in 1.65K pellet, suggesting that the DEX-packaging vesicles we collected were mainly MVs. Related to Fig. 1c. (**B**) HPLC analysis of the quantity of DEX in MVs. Drug contents of MVs were calculated according to the standard curve. Related to Fig. 1d. (**C**) The stability of MV-DEX was analyzed under various conditions. MV-DEX were suspended in PBS and treated with different conditions, including put in RT or 37 ℃ for a week, suspended in pH 8.5 or 5.5 solution, dealt with SDS (0.1%, 0.5%) or proteinase K (0.5 μg/L) or Triton-100 (0.1%) for 10 min. MVs were then recollected and counted by NTA. Data are presented as mean ± SD, n=3 independent experiments.

Supplementary Figure 2

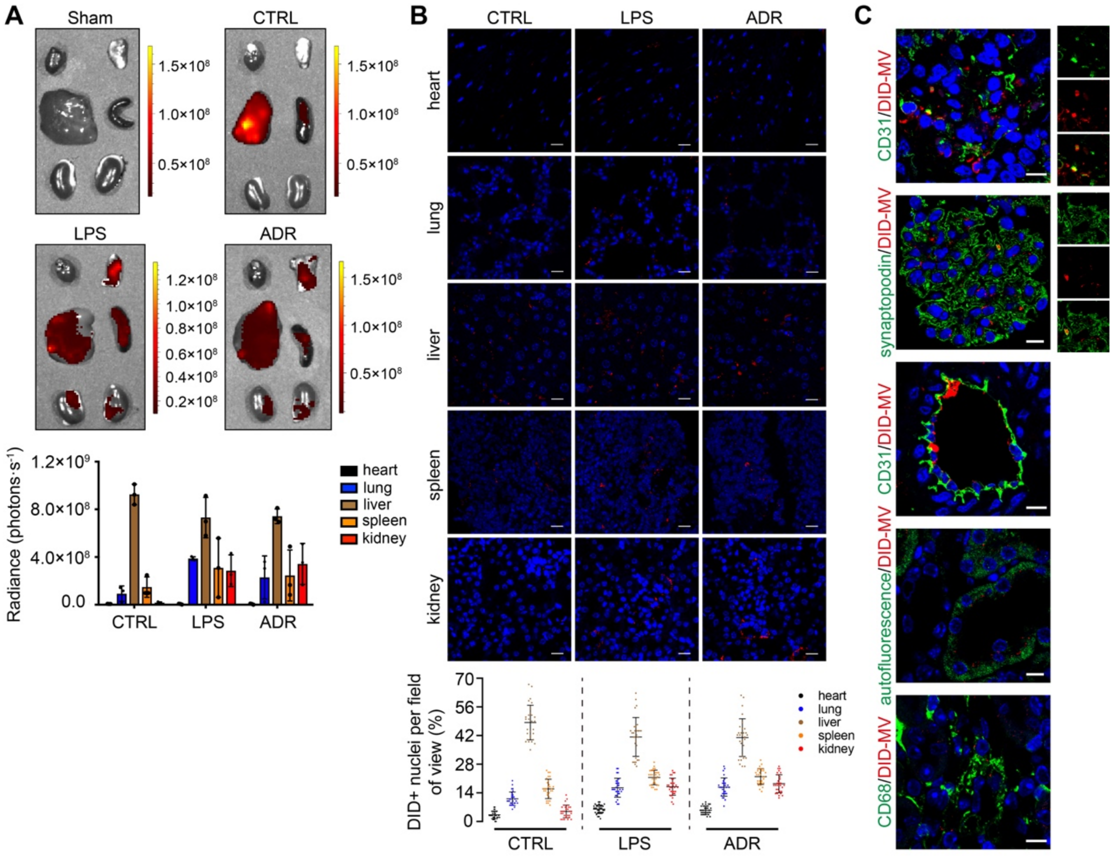

Fig.2 Tissue distribution of DEX-packaging MVs.

(**A**) Imaging of the indicated organs for detection of DID-labeled MV-DEX 24 h after i.p. injection. n=3 mice. (**B**) Representative micrographs of the indicated organs of mice injected with DID-labelled MV-DEX. n=6 mice. scale bar, 25 µm. Data are presented as mean ± SD. (**C**) MVs were detectable within the endothelial cells (CD31), podocytes (synaptopodin), tubular cells and macrophages (CD68). scale bar, 10 µm.

Supplementary Figure 3

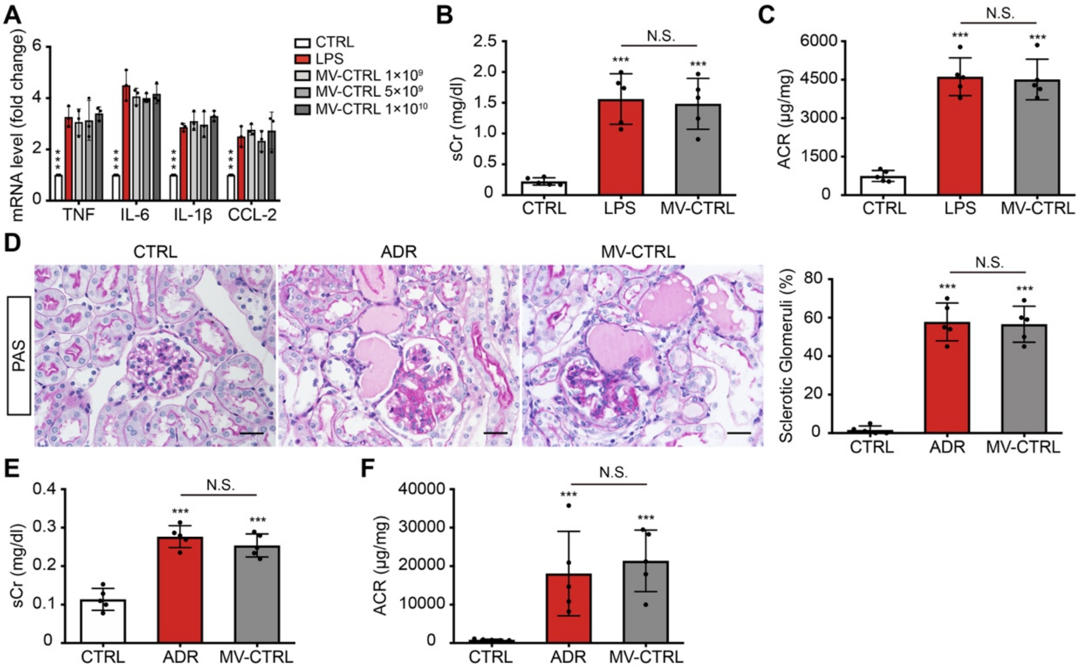

Fig.3 Drug-free MVs derived from RAW macrophages showed no therapeutic effects.

(A) GECs were treated with different doses of drug-free MVs for 12 h, and then inflammatory cytokine (TNF-α, IL-6, IL-β and CCL-2) mRNA expression were detected by real-time PCR. n=3 independent experiments. *** $p<0.001$ vs. LPS-treated group, one-way ANOVA. **(B, C)** Serum creatinine and albuminuria (urine albumin-to-creatinine ratio) in the LPS-treated mice at 2 days. **(D)** Representative images of PAS staining. scale bars, 25 μm. **(E, F)** Serum creatinine and albuminuria (urine albumin-to-creatinine ratio) in the ADR-treated mice at 3 weeks. n=5 mice per group. The amount of MVs-CTRL injected was about 1.5×10^{10}. Data are presented as mean ± SD, *** $p<0.001$ vs. CTRL mice, N.S., not significant, one-way ANOVA.

Supplementary Figure 4

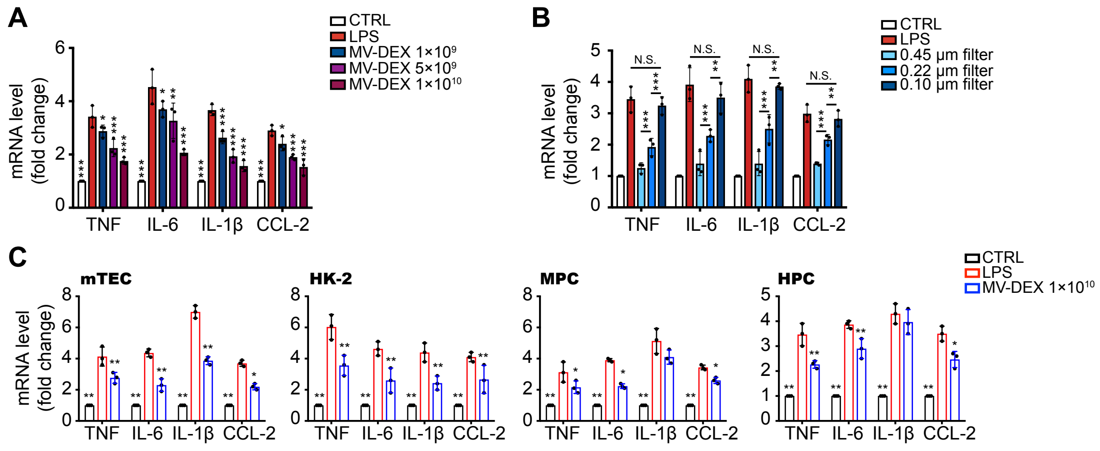

Fig.4 The anti-inflammatory efficacy of MV-DEX in vitro.

(**A**) GECs were treated with different doses of MV-DEX for 12 h, and then inflammatory cytokine (TNF-α, IL-6, IL-β and CCL-2) mRNA expression were detected by real-time PCR. (**B**) MV-DEX were suspended in PBS and were filtered by 0.45 μm, 0.22 μm, or 0.1 μm filter to reduce MVs, respectively. Real-time PCR analysis of the inflammatory cytokine mRNA expression. (**C**) In vitro inflamed mouse tubular cells (mTEC), human proximal tubular cells (HK-2), mouse podocytes (MPC) and human podocytes (HPC) were treated with 1×10^{10} MV-DEX, respectively. The anti-inflammatory efficacy was analyzed by real-time PCR. Data are presented as mean ± SD. n=3 independent experiments. NS, not significant, * p<0.05, ** p<0.01, *** p<0.001, one-way ANOVA.

Supplementary Figure 5

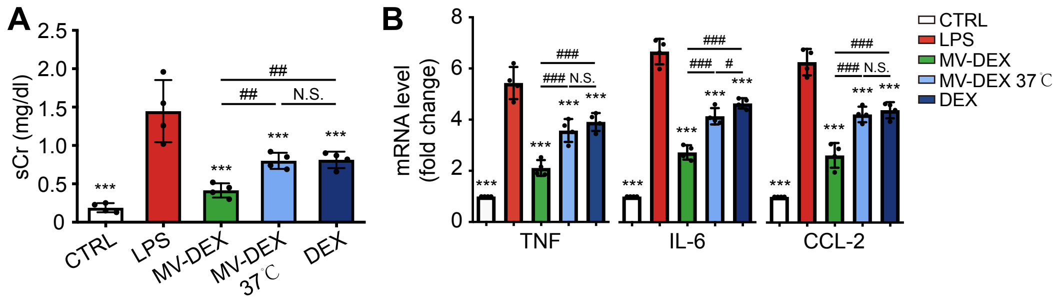

Fig.5 Effect of MV destruction on the anti-inflammatory efficacy of MV-DEX.

To determine whether MV-mediated drug delivery was responsible for this enhancement in vivo, we stored the MV-DEX and free DEX at 37 °C for 10 days to destroy most MVs, whereas the drug concentration between two groups remained the same. LPS-induced nephropathy and the treatment protocol was described in detail in the Methods. Serum creatinine (**A**) and cytokine (TNF-α, IL-6, IL-β and CCL-2) mRNA expression levels (**B**) were analyzed. n=4 mice per group. NS, not significant, *** p<0.001, # p<0.05, ## p<0.01, ### p<0.001, one-way ANOVA.

Supplementary Figure 6

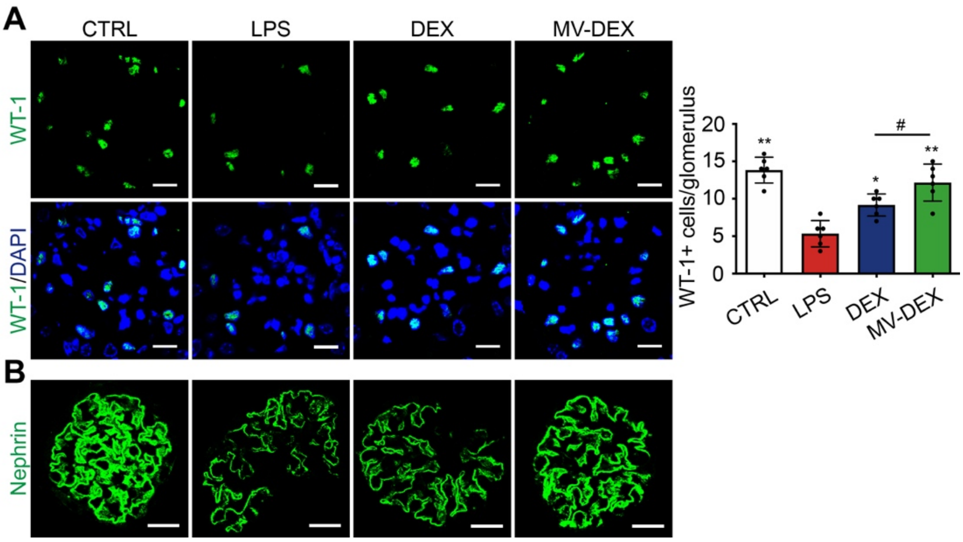

Fig.6 MV-DEX attenuate podocyte injury in LPS-treated mice.

Representative confocal microscopic images showing the expressions of WT-1 (**A**) and nephrin (**B**) in the kidney from different groups of mice. The number of WT-1 positive nuclei was determined in 50 sequential glomeruli per mouse. scale bar, 25 μm. n=6 mice per group. Data are presented as mean ± SD, * p<0.05, ** p<0.01 vs. LPS-treated mice, # p<0.05, one-way ANOVA.

Supplementary Figure 7

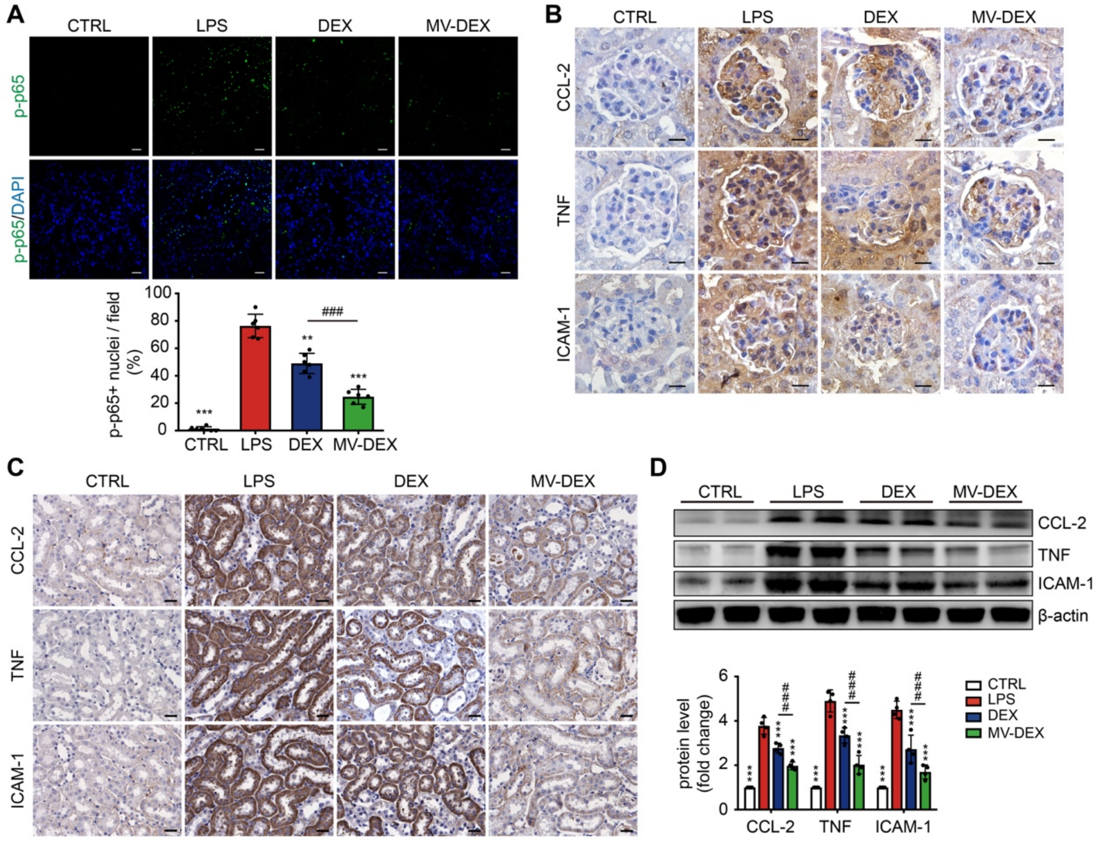

Fig.7 Enhanced anti-inflammatory efficacy of MV-DEX in LPS-treated mice.

(**A**) Representative confocal microscopic images of kidney sections stained with p-p65. The ration of p-p65 positive nuclei was determined in 8 representative fields per mouse. n=6 mice per group. (**B, C**) Immunostaining of CCL-2, TNF-α and ICAM-1 both in glomeruli and tubulointerstitium. (**D**) Expression of CCL-2, TNF-α and ICAM-1 in renal cortex tissue lysates. scale bar, 25 μm. n=4 mice per group. Data are presented as mean ± SD, ** p<0.01, *** p<0.001 vs. LPS-treated mice, ### p<0.001, one-way ANOVA.

Supplementary Figure 8

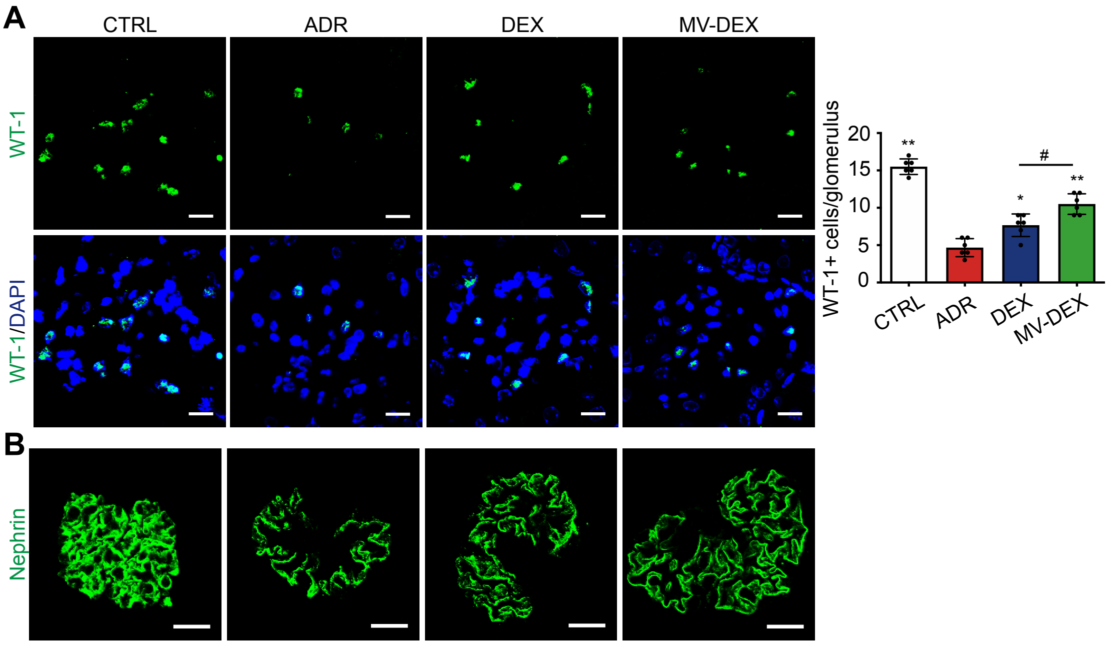

Fig.8 MV-DEX attenuate podocyte injury in ADR-treated mice.

Representative confocal microscopic images showing the expressions of WT-1 (**A**) and nephrin (**B**) in the kidney from different groups of mice. The number of WT-1 positive nuclei was quantified as described. scale bar, 25 μm. n=6 mice per group. Data are presented as mean ± SD, * p<0.05, ** p<0.01 vs. ADR-treated mice, # p<0.05, one-way ANOVA.

Supplementary Figure 9

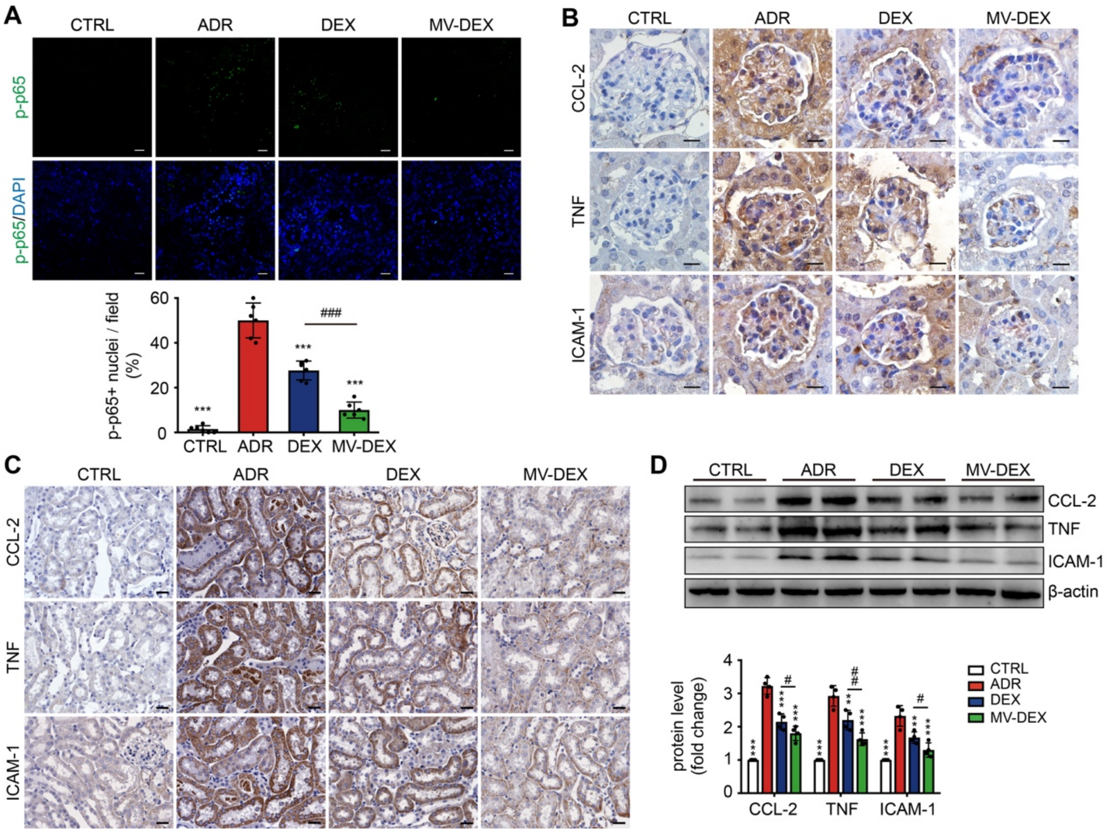

Fig.9 Enhanced anti-inflammatory efficacy of MV-DEX in ADR-treated mice. (**A**) Representative confocal microscopic images and quantification of kidney sections stained with p-p65. n=6 mice per group. (**B, C**) Immunostaining of CCL-2, TNF-α and ICAM-1 both in glomeruli and tubulointerstitium. (**D**) Expression of CCL-2, TNF-α and ICAM-1 in renal cortex tissue lysates. scale bar, 25 μm. n=4 mice per group. Data are presented as mean ± SD, ** $p<0.01$, *** $p<0.001$ vs. ADR-treated mice, # $p<0.05$, ## $p<0.01$, ### $p<0.001$, one-way ANOVA.

Supplementary Figure 10

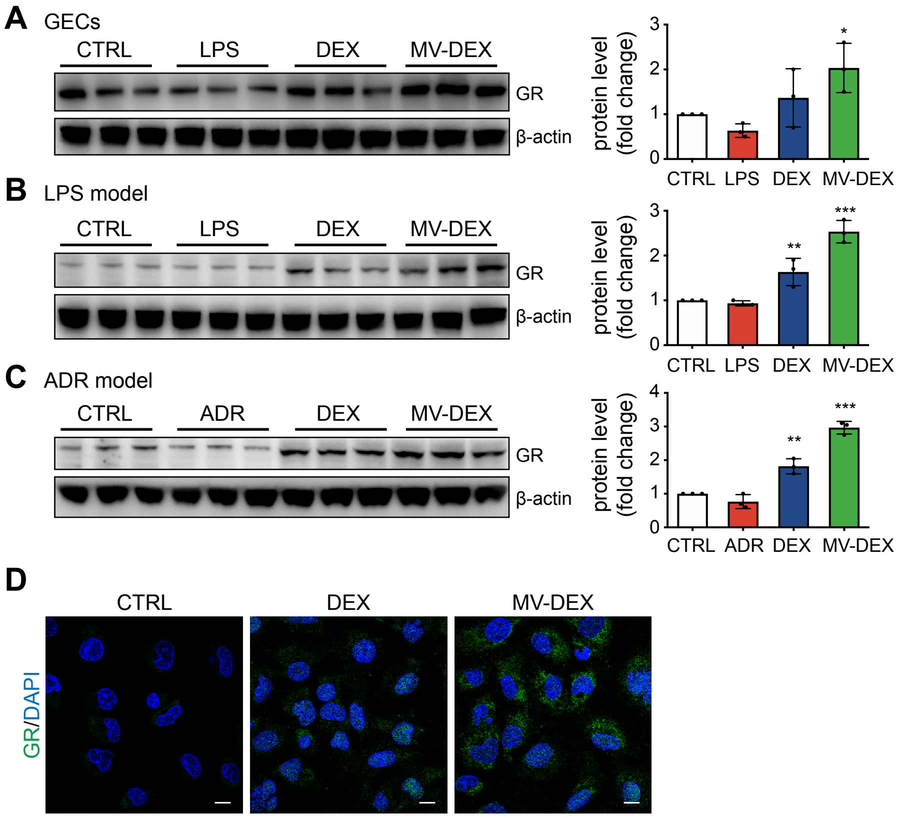

Fig.10 MV-DEX increase the expression of GR in vivo and in vitro.
Expression of GR in GECs (**A**), LPS-treated mice (**B**) and ADR-treated mice (**C**). n=3 mice per group. Data are presented as mean ± SD, * $p<0.05$, ** $p<0.01$, *** $p<0.001$ vs. control group, one-way ANOVA. (**D**) GECs were administered with free DEX or MV-DEX (with 5 μmol/L DEX) for 12 h. Representative confocal microscopic images of GECs stained with GR. scale bar, 10 μm.

Supplementary Figure 11

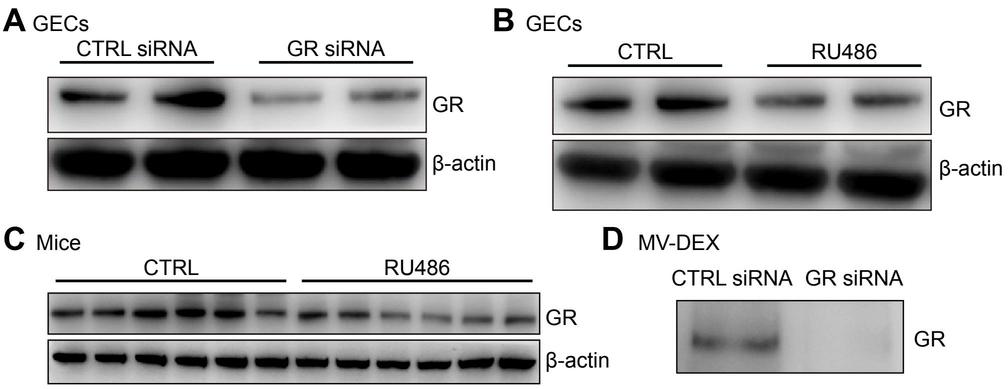

Fig.11 Downregulation of GR in vitro and in vivo.

Downregulation of GR was verified by Western blot analysis. (**A**) GECs were transfected with control siRNA or GR siRNA for 12 h. (**B**) GECs were treated with vehicle or RU486 for 12 h. (**C**) Mice were treated with vehicle or RU486 for 7 days. (**D**) MVs were isolated from control siRNA- or GR siRNA -transfected GECs.

Supplementary Figure 12

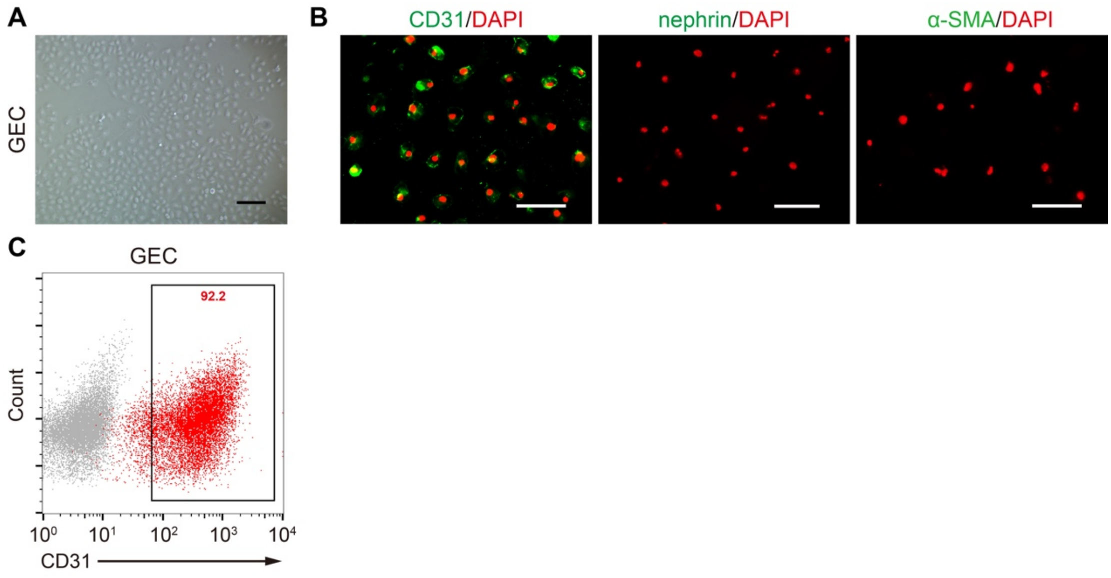

Fig.12 Characterization of primary glomerular endothelial cells (GECs).
After 7 days of culture, GECs were observed by a microscope (**A**) and were identified by immunofluorescence staining (**B**), which revealed that GECs were positive for CD31 (endothelial cells) and negative for nephrin (podocyte) or α-SMA (mesangial cell). (**C**) Flow cytometry analysis showed more than 90% of the cells were positive for CD31.

Supplementary Figure 13

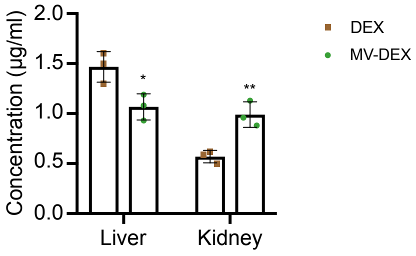

Fig.13 HPLC analysis of the DEX concentration in liver and kidney.
ADR- induced nephropathy and the treatment protocol was described in detail in the Methods. The liver and kidney were rinsed, weighed, and homogenized in two volumes of lysis buffer (based on tissue weight). DEX concentration in tissue homogenates was determined by HPLC. n=3 mice per group. Data are presented as mean ± SD, * $p<0.05$, ** $p<0.01$, two-tailed t-test.

Supplementary Figure 14

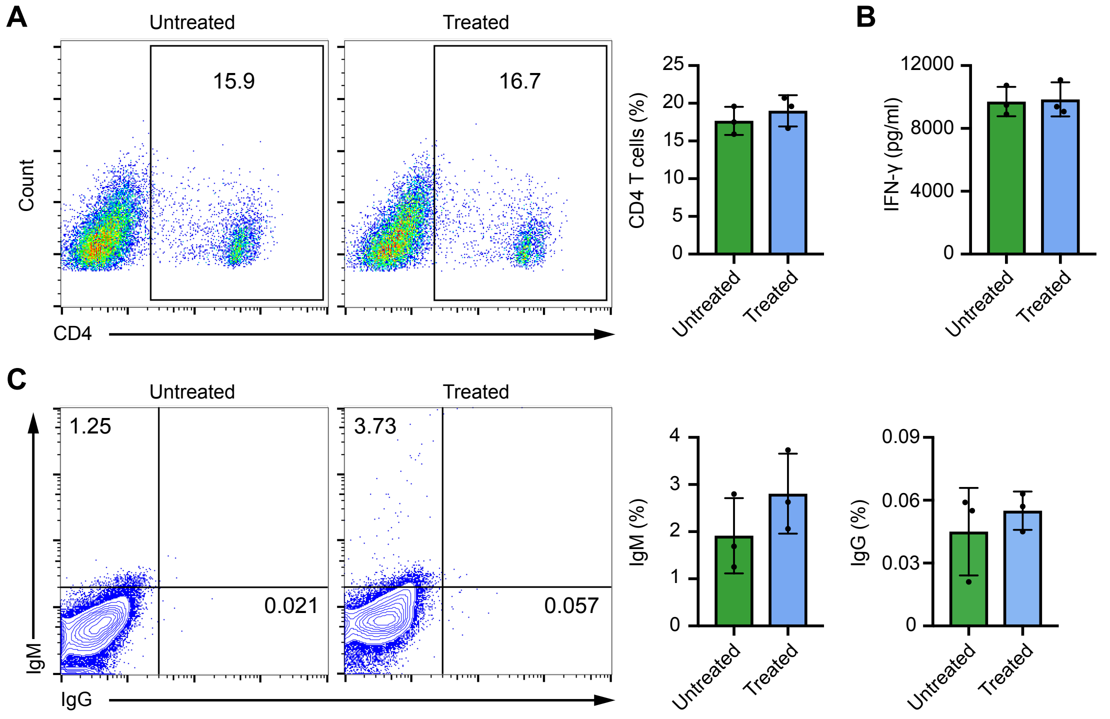

Fig.14 Immunogenicity of MV-DEX.

(**A**)(**B**) Mixed lymphocyte reaction assay was performed to evaluate the immunogenicity of MV-DEX. Splenocytes from BALB/C mice were prepared as stimulator cells and were exposed to ultraviolet irradiation. Splenocytes from ADR-treated C57/B6 mice exposed or not to MV-DEX treatment were used as responder cells (treatment protocol of MV-DEX was described in detail in the Methods). The BALB/C splenocytes were mixed with C57/B6 splenocytes at a ratio of 1:10 in culture medium (RPMI1640 supplemented with 10% FBS). After 6 days of coculture, the proliferation of CD4 T cells was analyzed by flow cytometry (**A**) and the IFN-γ expression was quantified by ELISA (**B**). There was no significant difference in CD4 T cell proliferation between the two groups. (**C**) Flow cytometry analysis profiles of IgM- and IgG-positive MV-DEX, previously incubated with the serum of mice untreated or treated with MV-DEX, which showed no significant elevation of autologous antibody compared to the untreated group. Data are presented as mean ± SD, two-tailed t-test.

Supplementary Figure 15

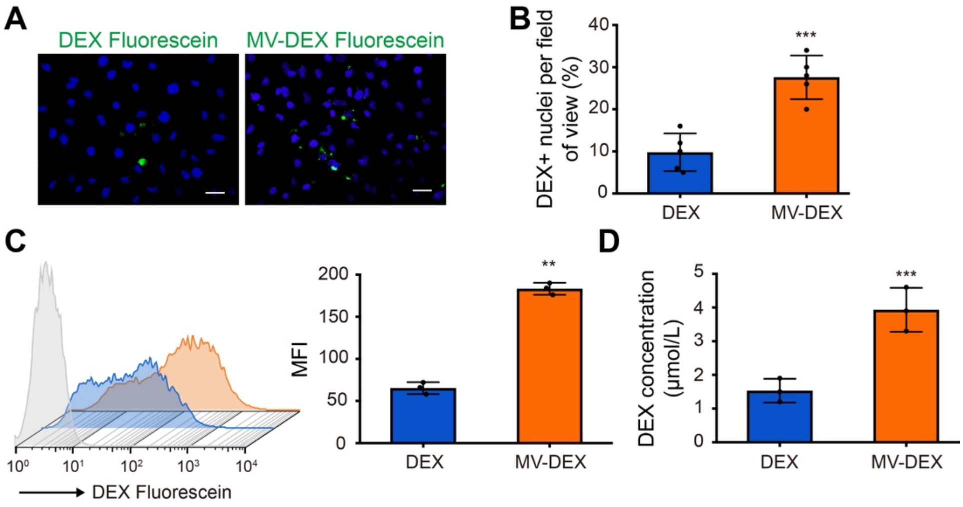

Fig.15 Macrophage MVs facilitate the entry of DEX into the cells.

GECs were treated with fluorescein-DEX and MVs-fluorescein-DEX at the same drug concentration for 12 h. (with 10 μmol/L fluorescein-DEX) (**A, B**) Representative microscopic images and quantification of fluorescein-DEX in GECs. (**C**) fluorescein-DEX positive cells were detected by flow cytometry. (**D**) The drug concentration in GECs (5×10^8 cells) were analyzed by HPLC. scale bar, 25 μm. Data are presented as mean ± SD, ** p<0.01, *** p<0.001, two-tailed t-test.

Supplementary Figure 16

Uneditied gel for figure 2C

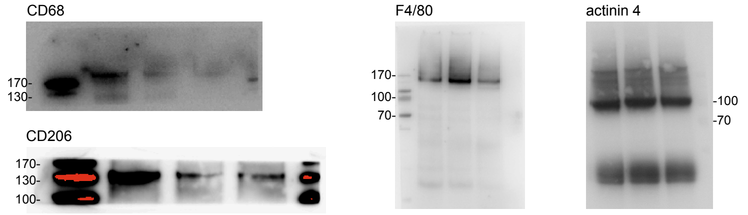

Uneditied gel for figure 4B

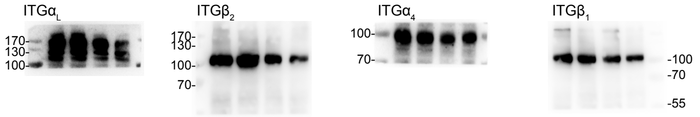

Uneditied gel for figure 4C

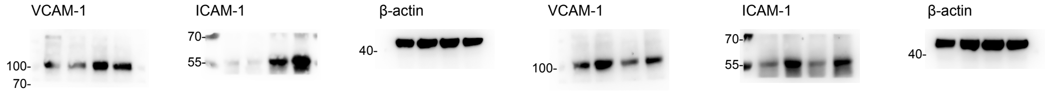

Uneditied gel for figure 5F

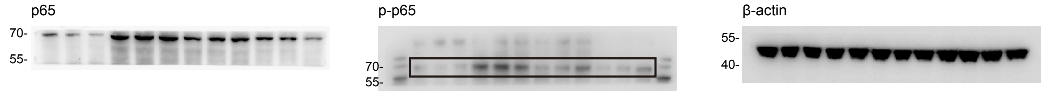

Uneditied gel for figure 6H

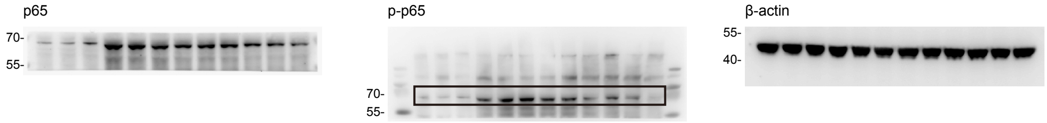

Uneditied gel for figure 7J

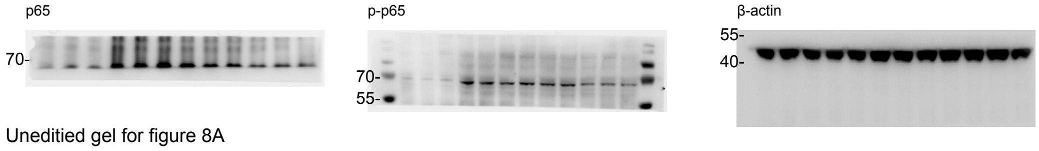

Uneditied gel for figure 8A

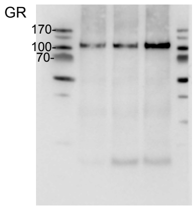

Uneditied gel for figure 8I

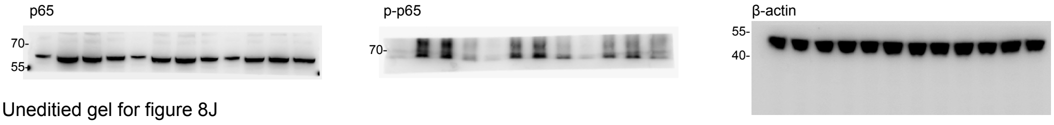

Uneditied gel for figure 8J

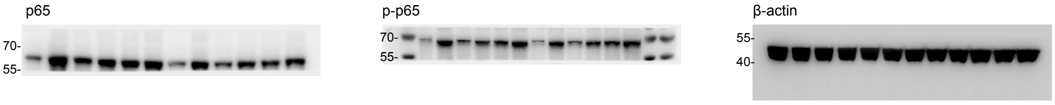

Uneditied gel for supplementary figure 1A

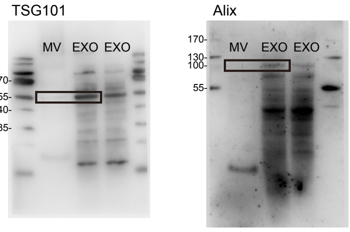

Uneditied gel for supplementary figure 6D

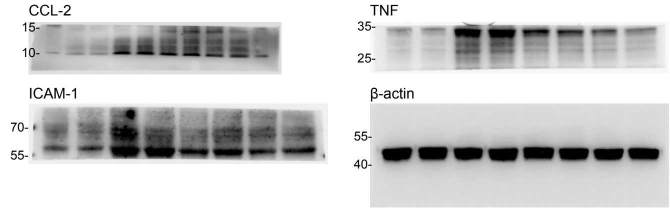

Uneditied gel for supplementary figure 8D

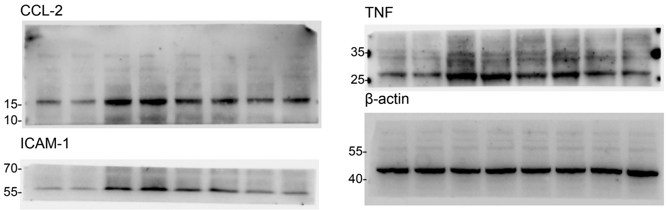

Uneditied gel for supplementary figure 9A-C

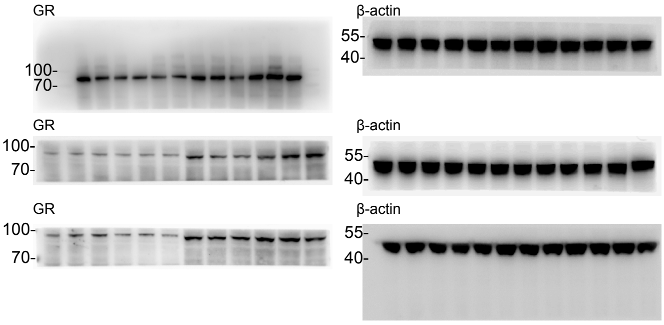

Uneditied gel for supplementary figure 10A-B

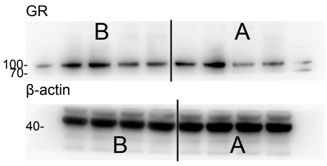

Uneditied gel for supplementary figure 10C

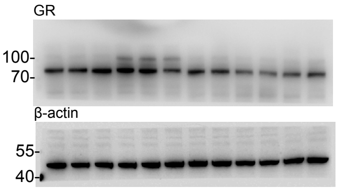

Uneditied gel for supplementary figure 10D

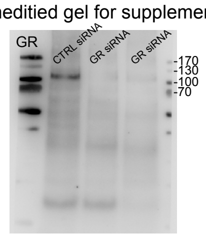

Supplement: Supplementary file 1 — Supplementary figures and tables. [file thnov09p4740s1.pdf]
